# Human Epidermal Growth Factor Receptor-3 Expression Is Regulated at Transcriptional Level in Breast Cancer Settings by Junctional Adhesion Molecule-A via a Pathway Involving Beta-Catenin and FOXA1

**DOI:** 10.3390/cancers13040871

**Published:** 2021-02-19

**Authors:** Rodrigo G. B. Cruz, Stephen F. Madden, Cathy E. Richards, Sri HariKrishna Vellanki, Hanne Jahns, Lance Hudson, Joanna Fay, Naoimh O’Farrell, Katherine Sheehan, Karin Jirström, Kieran Brennan, Ann M. Hopkins

**Affiliations:** 1 Department of Surgery, Royal College of Surgeons in Ireland, Beaumont Hospital, Dublin 9, Ireland; rodrigobernardo@rcsi.ie (R.G.B.C.); catherinerichards@rcsi.ie (C.E.R.); shkvellanki@rcsi.ie (S.H.V.); lhudson@rcsi.com (L.H.); k.brennan@ucd.ie (K.B.); 2 Data Science Centre, Royal College of Surgeons in Ireland, Dublin 2, Ireland; stephenmadden@rcsi.ie; 3 Pathobiology Section, UCD School of Veterinary Medicine, University College Dublin, Dublin 4, Ireland; hanne.jahns@ucd.ie; 4 Department of Pathology, Royal College of Surgeons in Ireland, Beaumont Hospital, Dublin 9, Ireland; joannafay@rcsi.ie (J.F.); naoimhofarrell@rcsi.ie (N.O.); ksheehan@rcsi.ie (K.S.); 5 Department of Clinical Sciences Lund, Division of Oncology and Therapeutic Pathology, Lund University, SE 221 85 Lund, Sweden; karin.jirstrom@med.lu.se

**Keywords:** JAM-A, HER2, HER3, FOXA1, β-catenin, breast cancer, tight junction, transcription factor, anti-HER2 therapies, HER2-targeted therapies, drug resistance

## Abstract

**Simple Summary:**

Signaling from the human epidermal growth factor receptor (HER) family of proteins increases in many cancers, including breast. HER2-high breast cancers are successfully treated with anti-HER2 therapies, but these drugs are limited by the fact that patients frequently develop resistance to them. One common mechanism by which resistance develops is when tumors acquire high levels of a family member called HER3. We had previously shown that a protein called JAM-A regulates the level of HER2 in breast cancer cells, and is associated with the development of resistance to HER2-targeted therapies. In this study we show for the first time that JAM-A levels also regulate those of HER3. Using breast cancer cell and tissue models and culminating in patient tissue material, we provide evidence that JAM-A regulates HER3 expression via a pathway involving the transcription factors β-catenin and FOXA1. We suggest that JAM-A merits future investigation as a novel drug target for its potential to reduce HER3 tumorigenic signaling and to offset the development of resistance to HER2-targeted therapies.

**Abstract:**

The success of breast cancer therapies targeting the human epidermal growth factor receptor-2 (HER2) is limited by the development of drug resistance by mechanisms including upregulation of HER3. Having reported that HER2 expression and resistance to HER2-targeted therapies can be regulated by Junctional Adhesion Molecule-A (JAM-A), this study investigated if JAM-A regulates HER3 expression. Expressional alteration of JAM-A in breast cancer cells was used to test expressional effects on HER3 and its effectors, alongside associated functional behaviors, in vitro and semi-in vivo. HER3 transcription factors were identified and tested for regulation by JAM-A. Finally a patient tissue microarray was used to interrogate connections between putative pathway components connecting JAM-A and HER3. This study reveals for the first time that HER3 and its effectors are regulated at gene/protein expression level by JAM-A in breast cancer cell lines; with functional consequences in in vitro and semi-in vivo models. In bioinformatic, cellular and patient tissue models, this was associated with regulation of the HER3 transcription factor FOXA1 by JAM-A via a pathway involving β-catenin. Our data suggest a novel model whereby JAM-A expression regulates β-catenin localization, in turn regulating FOXA1 expression, which could drive HER3 gene transcription. JAM-A merits investigation as a novel target to prevent upregulation of HER3 during the development of resistance to HER2-targeted therapies, or to reduce HER3-dependent tumorigenic signaling.

## 1. Introduction

Members of the human epidermal growth factor receptor (HER) family of tyrosine kinases have long been implicated in breast cancer development and progression. ErbB2/HER2 has received particular attention, its amplification/overexpression having defined a genomic subtype of breast cancer [1] and inspired a generation of HER2-targeted therapies [2]. However ErbB3/HER3 is emerging as a player of significant interest. Ligand-activated HER3 can heterodimerize with all members of the EGFR family (EGFR/HER1, ErbB2/HER2, ErbB4/HER4), promoting tyrosine phosphorylation to activate pro-proliferative and anti-apoptotic signaling pathways via mitogen-activated protein kinase (MAPK) and phosphatidylinositol 3-kinase (PI3K)/AKT [3,4]. HER3 overexpression has been reported in ~18% of breast cancer patients, and is linked with poor prognosis and reduced survival [5]. Importantly, HER3 has also been implicated in the development of resistance to anti-HER2 targeted therapies [6,7]; reflecting their frequent co-expression [8] and the likelihood of compensatory dimerization between HER3 and HER1 or HER4.

In contrast to HER2, HER3 overexpression usually results from increased gene transcription rather than gene amplification [9], but little is known about the mechanisms of regulation. However as resistance to HER2-targeted therapeutic antibodies is common in patients, the identification of new upstream targets would be an asset. In this context, the adhesion protein Junctional Adhesion Molecule-A (JAM-A) shows promise [10,11,12,13]. Notwithstanding several physiological roles for normal-level JAM-A expression in various cell types [14], we [11,13] and others [15,16,17,18,19] have linked high JAM-A expression with aggressive disease and poor outcome in patients with several solid tumors including breast. JAM-A antagonism has also been shown to inhibit tumor growth in a murine xenograft breast cancer model [17]; and JAM-A abrogation in mice has been linked to the induction of apoptosis and reduced breast cancer progression [19].

In the context of breast cancer, we have previously reported correlations between JAM-A and HER2 expression in breast cancer cells and patient tissues, and provided evidence that JAM-A expression uni-directionally regulates that of HER2 [13]. We have also shown a functional link between JAM-A expression and the development of resistance to HER2-targeted therapies in breast cancer patients [20]. In the current study we therefore questioned whether JAM-A expression regulated that of HER3 and its signaling effectors. We present novel data that JAM-A regulates HER3 at gene expression level via a pathway involving β-catenin and FOXA1. Importantly, functional in vitro and semi-in vivo data have been supported by evidence of a linear pathway between JAM-A, β-catenin, FOXA1 and HER3 expression in tissues from patients with invasive breast cancer. Collectively, these novel data suggest that JAM-A merits future investigation as a target to overcome HER3 tumorigenic signaling and resistance to HER2-targeted therapies in breast cancer patients. 

## 2. Materials and Methods

### 2.1. Cell Culture

MCF7 and MDA-MB-231 cells were purchased from the American Tissue Culture Collection (Manassas, VA, USA). SK-BR-3 cells were a kind gift of Drs. Alex Eustace and Norma O’Donovan (Dublin City University, Dublin 9, Ireland) [21]. MCF7 cells were maintained in Minimum Essential Medium Eagle medium supplemented with 10% fetal bovine serum, 50 U/mL penicillin, 50 µg/mL streptomycin, 2 mM L-glutamine and 1% non-essential amino acids (Sigma-Aldrich, Dharmstadt, Germany). SK-BR-3 cells were maintained in RPMI-1640 medium and MDA-MB-231 cells in Dulbecco’s Modified Eagle Medium, both supplemented as described before and maintained at 37 °C and 5% CO_2_ in a humidified environment. MCF7, SK-BR-3 and MDA-MB-231 cells overexpressing JAM-A (MCF7-JAM+, SK-BR-3-JAM+ and MDA-MB-231-JAM+) were generated by transfection of full-length human JAM-A DNA in a pcDNA3 plasmid (a kind gift from Prof. Charles Parkos, University of Michigan, Ann Arbor, MI, USA) [22] into cells and subsequent selection with G418. Cells stably expressing the empty vector pcDNA3 were used as controls. Throughout the manuscript, we have referred to JAM-A-overexpressing cells as “cell line-JAM+” and control empty vector-overexpressing cells as “cell line” (e.g., “MCF7-JAM+” versus “MCF7”). All cell lines were confirmed as mycoplasma-free on a quarterly basis using MycoAlert detection kit (Lonza, Basel, Switzerland). Cell lines were sequenced once-yearly via Short Tandem Repeat genotyping to confirm their identity (Source BioScience, Nottingham, UK).

### 2.2. siRNA Transfection

Cells were transiently transfected with siRNAs using DharmaFECT 1 (Dharmacon, Lafayette, IN, USA) according to manufacturer protocols. A pool of non-targeting siRNAs (siNEG) was used as a negative control (siGENOME Non-Targeting siRNA Pool #1, Dharmacon). JAM-A silencing was carried out using two siRNA sequences in a pool (F11R siRNA NM_016946-SASI_Hs01_00049785, Sigma Aldrich and JAM-A siRNA designed by us and manufactured by Dharmacon—sense sequence CGGGGGUCGCAGGAAUCUGUU). JAM-A silencing results were confirmed using different siRNA sequences as described in Figure S1. HER3, HER2, FOXA1 and β-catenin gene silencing was carried out using ERBB3 siRNA (L-003127-00-0005, Dharmacon), ERBB2 siRNA (L-003126-00-0005, Dharmacon), FOXA1 siRNA (L-010319-00-0005, Dharmacon) and CTNNB1 siRNA (L-003482-00-0005, Dharmacon) consisting of smartpools of four siRNA sequences each. All siRNAs were incubated at final concentration 25 nM for 72 h.

### 2.3. Protein Electrophoresis and Western Immunoblotting

Whole-cell lysates were prepared in lysis buffer (0.1 M KCl, 2.5 mM NaCl, 3.5 mM MgCl_2_, 10 mM HEPES, 1% Triton-X100 and 1× protease/phosphatase inhibitors) and equivalent protein concentrations (as determined by bicinchinoic acid protein assays, ThermoFisher, Waltham, MA, USA) separated by SDS–polyacrylamide gel electrophoresis using Bio-Rad Mini-Protean III gel systems (Bio-Rad Laboratories, Hertfordshire, UK). Proteins were wet-transferred to nitrocellulose or PVDF membranes and immunoblotted with antibodies to human JAM-A (mouse anti-JAM-1, 612120, BD Biosciences, San Jose, CA, USA), FoxA1/HNF3α (D7P9B) (58613, Cell Signaling, Danvers, MA, USA), HER3/ErbB3 (D22C5) (12708, Cell Signaling), AKT (pan) (40D4) (2920, Cell Signaling), Phospho-AKT (Ser473) (9271, Cell Signaling), p44/42 MAPK (Erk1/2) (L34F12, Cell Signaling), Phospho-p44/42 MAPK (Erk1/2) (Thr202/Tyr204) (4370, Cell Signaling), β-catenin (D10A8) (8480, Cell Signaling), beta-Actin (ab8227, Abcam, Cambridge, UK) or Lamin A/C (4C11) (4777, Cell Signaling). This was followed by incubation with HRP-linked secondary antibodies (goat anti-rabbit IgG, 7074, Cell Signaling; goat anti-mouse IgG, A9044, Sigma-Aldrich). Following incubation with Western Lightning Plus ECL (PerkinElmer Waltham, MA, USA), blots were imaged on the ChemiDoc XRS+ system (Bio-Rad), and densitometry analysis carried out using ImageJ [23]. Band intensity for each protein was normalised to that of its own loading control (either actin or Lamin A/C, as appropriate), and densitometric graphs represent the averaged results of *n* = 3 experiments. Raw, uncropped blots are shown as Supplementary Data in Document S1.

### 2.4. Quantitative Reverse Transcription PCR

RNA was extracted from cells using TRI-Reagent (T9424, Sigma-Aldrich), according to the manufacturer’s instructions. cDNA was obtained using QuantiTect Reverse Transcription Kit (Qiagen, Venlo, The Netherlands) and qRT-PCR performed using LightCycler-480 SYBR Green I Master Mix (04707516001, Roche, Basel, Switzerland) on a Roche LightCycler-480 instrument. RNA expression was evaluated by the relative qPCR analysis method using RPLP0 as a reference housekeeping gene to standardize and compare Ct values between different treatments. Data analysis was performed using the following calculations: Average Ct values for all gene replicates, Delta Ct value between gene of interest and housekeeping gene for each experiment, Average Delta Ct values between experiments (replicates), Delta-Delta Ct values (Delta Ct experiment − Delta Ct control) and finally Fold Change [2^(−Delta Delta Ct)]. Levels of mRNA were assessed in all cell lines using the following primers:

JAM-A: 

Fwd 5′-CTCTCAGTCCCCTCGCTGTA-3′; Reverse AATGCCAGGGAGCACAACAG

HER3: 

Fwd 5′-CACAGATGGTCTTGGTCAATGTC-3′; Reverse CACAGATGGTCTTGGTCAATGTC

FOXA1: 

Fwd 5′-AGGGCTGGATGGTTGTATTG-3′; Reverse GCTCGTAGTCATGGTGTTCAT

β-catenin: 

Fwd 5′-CCTTCAACTATTTCTTCCATGCG-3′; Reverse CTAGTTCAGTTGCTTGTTCGT G

RPLP0:

Fwd 5′-GGCAGCATCTACAACCCTGA-3′; Reverse AACATTGCGGACACCCTCC

### 2.5. Cell Viability Assays

Cellular viability/proliferation was measured by performing 3-(4,5-dimethylthiazol-2-yl)-2,5-diphenyl tetrazolium bromide (MTT, Sigma-Aldrich) assays. Specifically, 1500–6000 cells were seeded in triplicate wells of 96-well plates, treated and incubated for periods as indicated in figure legends. MTT reagent was added (final concentration 0.5 mg/mL) and incubated in the dark for 5 h at 37 °C. Following solubilization with DMSO, absorbance was measured at 560 nm using a VICTOR™ X3 Multilabel Plate Reader (Perkin Elmer, Waltham, MA, USA).

### 2.6. Clonogenic Assays

Three thousand cells were seeded per well in 6-well plates. Gene silencing experiments (as described earlier) were performed on day 3 and repeated 72 h later to ensure that gene expression levels were continuously suppressed. Following 9 days of incubation, colonies were fixed in 100% methanol for 5 min and stained with 0.5% crystal violet for 40 min at RT.

### 2.7. Protein-DNA Binding Assays

Bioinformatics analysis was carried out in order to identify putative regions where the FOXA1 transcription factor bound in the proximal promoter of the HER3 gene in breast cancer cells. The platforms used were the Encyclopedia of DNA Elements (ENCODE) project (https://www.encodeproject.org/ (accessed on 10 May 2020)), TRANSFAC [24] and the TFFFIND search tool from the Piptools package [25]. Based upon this, DNA probes corresponding to the FOXA1-binding sequence in the HER3 promoter were constructed (Integrated DNA Technologies, Coralville, IA, USA) using the following sequences (putative FOXA1 binding sequences are underlined): 

Oligonucleotide 1:

5′-AGAAATATTCACATTCTGAGAGAAAATCCACCAAGTGAACCAACC-3′ 

Scrambled Control Oligonucleotide 1: 

5′-GACATAGAACTACCCAATGCAATCAGAAGTCACACGTCAAATAAT-3′ 

Oligonucleotide 2: 

5′-CCGGCTCCGGCTCCGATTGCAATTTGCAACCTCCGCTGCC-3′

Nuclear extracts were prepared from cells using EpiQuik™ Nuclear Extraction Kits (Epigentek, Farmingdale, NY, USA) according to the manufacturer’s instructions. To detect the binding of FOXA1 to oligonucleotide sequences representing the HER3 promoter in vitro, the EpiQuik™ General Protein-DNA Binding Assay Kit (Colorimetric) (Epigentek) was used according to the manufacturer’s instructions. A FOXA1/HNF3α (D7P9B; 58613, Cell Signaling) antibody was used to detect specific protein-DNA binding. Binding Activity = delta optical density (sample—blank) × sample dilution.

### 2.8. Double Immunofluorescence (IF) Labeling and Epifluorescence or Confocal Microscopy

Cells were plated at 30,000 cells per well on 13 mm round glass coverslips in 24-well plates and transiently transfected as described above. Cells were fixed with pure ethanol for 20 min at −20 °C and blocked with 5% goat serum. The cells were then incubated in primary antibody (JAM-A-sc-53623, Santa Cruz, Heidelberg, Germany; FoxA1-58613, Cell Signaling; β-catenin-8480, Cell Signaling) overnight at 4 °C. Cells were incubated in blocking buffer containing 2 μg/mL secondary antibody (either Alexa-Fluor-488 goat anti-mouse IgG, A32723 or Alexa-Fluor-568 goat anti-rabbit IgG, A11079, ThermoFisher) and then inverted onto slides using Vectashield mounting medium containing DAPI (H-1200, Vector Laboratories, Burlingame, CA, USA). Fluorescent micrographs were acquired at 40× magnification using a CKX41 epifluorescent microscope (Olympus, Hamburg, Germany) connected to a charge-coupled device color camera, with identical exposure settings used for directly-matched control versus test conditions (Cell B imaging software, Olympus, Hamburg, Germany); or on an LSM510-Meta confocal microscope (Carl Zeiss, Oberkochen, Germany) using 63× oil immersion lenses and identical thresholding for control versus test samples.

### 2.9. Immunohistochemistry (IHC)

Immunohistochemistry (IHC) for JAM-A (F11R monoclonal antibody, clone RM275-MAB14922, Abnova, Taipei, Taiwan), FoxA1 (58613, Cell Signaling), HER3 (12708, Cell Signaling) and β-catenin (8480, Cell Signaling) was carried out on serial sections of a tissue microarray (TMA) containing duplicate cores from a consecutive cohort of 144 patients diagnosed with invasive breast cancer at Skåne University Hospital, Malmö, Sweden, between 2001 and 2002 [26]. Staining was performed by the Beaumont Hospital/RCSI Histopathology Department using a Bond III automated IHC stainer (Leica, Wetzlar, Germany) following the manufacturer’s instructions and using Leica Biosystems reagents. The TMA was then independently scored by RGBC and a breast histopathologist (KS or NOF). Each core was assigned a value (0, 1+, 2+ or 3+) representing the overall intensity of FOXA1, HER3 and β-catenin expression. β-catenin was also scored in terms of its relative presence in the cell membrane, cytoplasm or nucleus. Membranous intensity of JAM-A protein expression was scored as follows: 0 (no staining or membrane staining in <10% of tumor cells); 1+ (faint/barely perceptible and incomplete membrane staining in >10% of tumor cells); 2+ (weak to moderate complete membrane staining in >10% of tumor cells); 3+ (strong complete membrane staining in >10% of tumor cells).

### 2.10. Semi-In Vivo Chick Embryo Xenograft Assay

Fertilized chicken eggs were purchased from Shannonvale Hatchery (Limerick, Ireland) and incubated at 37 °C. On day 3 a window was opened on the surface of the eggs and fluid withdrawn to lower the chorioallantoic membrane (CAM). On day 9, 2 × 10^6^ breast cancer cells resuspended in Matrigel were added to the CAM within a silica ring. Eggs with implanted xenografts were treated with control siNEG or JAM-A siRNA (25 nM final concentration) on days 10 and 12. On day 14, the embryos were sacrificed and tumor xenografts isolated by cutting the surrounding CAM 2–5 mm away from the silica ring. Tumor xenografts were fixed overnight at RT in 4% formaldehyde and washed with 70% ethanol the following day. Tumors were then paraffin-embedded and stained with hematoxylin/eosin or immunohistochemically stained for the proliferation marker Ki67. Ki67-positive and -negative cells were counted in 500 tumor cells per slide.

### 2.11. Statistical Analysis

Averaged data from triplicate experiments are presented and were graphed along with standard error of the mean (s.e.m.) values. Two-tailed, equal variance Student’s *t*-tests were used to determine statistical significance (* *p* < 0.05, ** *p* < 0.01, *** *p* < 0.001) between the indicated conditions. SPSS software (IBM, Armonk, NY, USA) was used to carry out statistical analysis comparing histopathological conditions and clinicopathological factors by χ^2^ test and Fisher’s two-tailed exact tests; and *p* < 0.05 was considered statistically significant.

## 3. Results

### 3.1. JAM-A Regulates mRNA and Protein Expression of HER3

**Figure 1:**
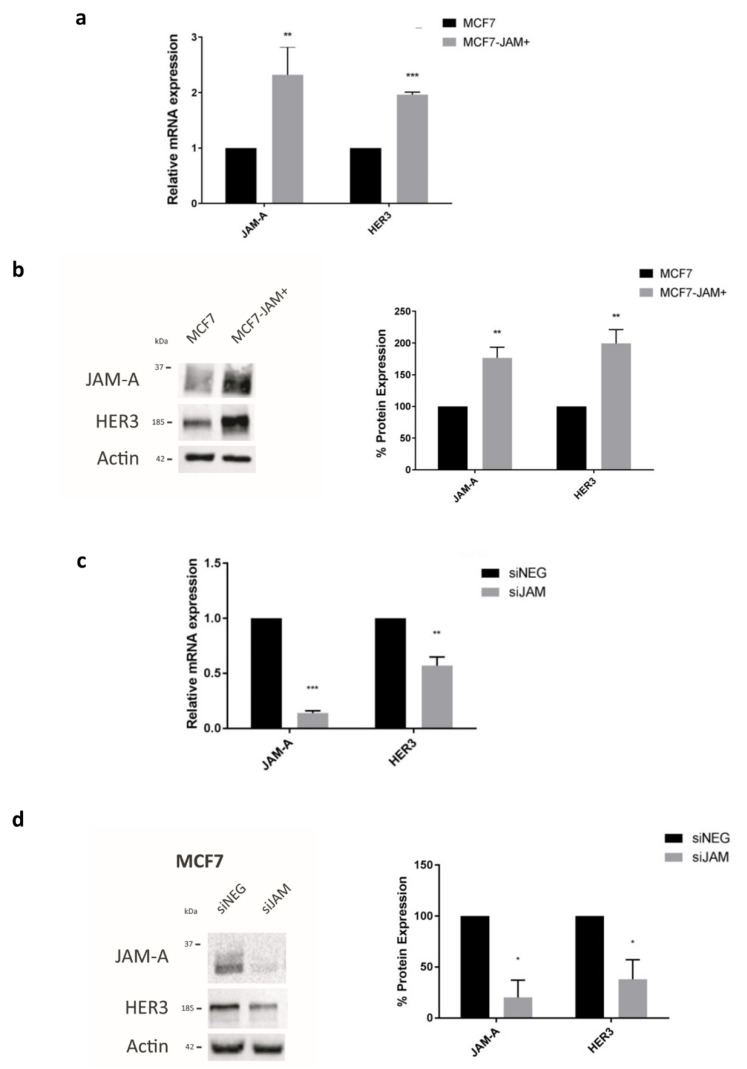
JAM-A levels influence mRNA and protein expression of HER3. RNA and protein from control MCF7 cells overexpressing empty vector (MCF7) and MCF7 cells overexpressing JAM-A (MCF7-JAM+), or MCF7 cells transfected with 25 nM control siRNA (siNEG) or a pool of JAM-A siRNA for 72 h were analyzed by qRT-PCR and immunoblotting. (**a**) Analysis of MCF7-JAM+ by RT-qPCR revealed significant upregulation of HER3 mRNA expression compared to that in wild type MCF7 cells. (**b**) Representative western blot image and densitometric analysis of JAM-A and HER3 normalized to actin revealed that overexpression of JAM-A in MCF7-JAM+ cells was associated with upregulation of HER3 protein expression compared to wild type MCF7 cells. (**c**) RT-qPCR revealed HER3 mRNA expression is significantly reduced after JAM-A knockdown compared to control conditions in MCF7 cells. (**d**) Representative western blot image and densitometric analysis of JAM-A and HER3 normalized to actin revealed that JAM-A gene silencing reduced HER3 protein expression compared to siNEG controls in MCF7 cells. Experiments were performed three times and data represent mean ± s.e.m, compared using two-tailed, equal variance Student’s *t*-tests, * *p* < 0.05, ** *p* < 0.01, *** *p* < 0.001.

In light of correlations between the expression of JAM-A and HER2 in breast tumors, and their links to poor patient outcome [11,13,20], we first set out to investigate links between JAM-A and other HER family members. Using online tools [27,28], we noted that high mRNA expression of both JAM-A and HER3 is observed in invasive breast carcinomas (Figure S2a,b respectively), and their coincident high expression is associated with reduced recurrence-free survival in breast cancer patients (Figure S2c). Since upregulation of HER3 [6] and alterations in JAM-A [20] have been associated with the development of resistance to HER2-targeted therapies in breast cancer patients, this study aimed to uncover mechanisms of JAM-A/HER3 crosstalk. Commencing at a reductionist level, MCF7 breast cancer cells genetically modified to overexpress JAM-A (MCF7-JAM+) versus pcDNA3 empty vector (MCF7) were used as a presumptive model for JAM-high cancers. JAM-A overexpression (*p* < 0.01) was associated with a significant increase in HER3 mRNA (Figure 1a, *p* < 0.001) and protein (Figure 1b) expression. Correspondingly, transient JAM-A gene silencing in MCF7 cells (versus control non-targeting siRNA; siNEG) reduced HER3 mRNA (Figure 1c, *p* < 0.01) and protein (Figure 1d, *p* < 0.05) expression. The effects of JAM-A overexpression and silencing on HER3 gene or protein expression were reproduced in SK-BR-3 and MDA-MB-231 breast cancer cells (overexpression in Figure S3, silencing in Figure S4). The relative protein expression levels of JAM-A and HER3 across the three cell line models are shown in Figure S5.

### 3.2. JAM-A or HER3 Gene Silencing Reduces Cell Viability and Levels of Survival Effector Proteins

**Figure 2:**
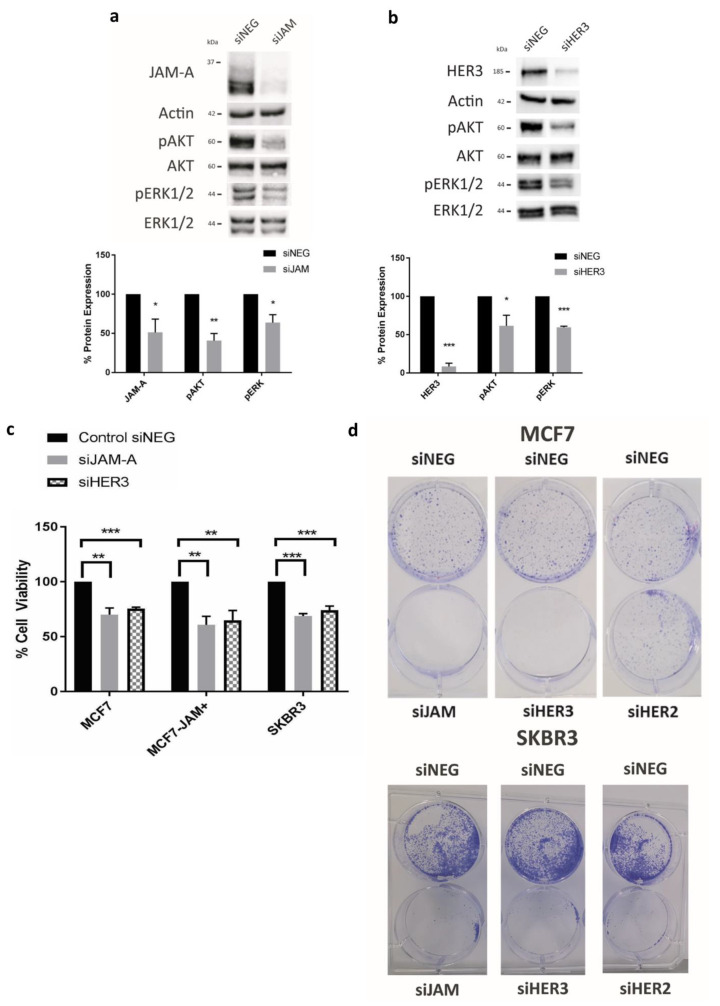
Silencing of JAM-A or HER3 reduces activation of AKT/ERK, cell viability and colony formation. Breast cancer cells were transfected with 25 nM control siRNA (siNEG) or a pool of siRNA targeting JAM-A, HER3 or HER2 and harvested at 72 h for immunoblotting or used in functional assays. Representative western blot image and densitometric analysis for pAKT protein expression normalized to AKT expression, pERK expression normalized to ERK expression and JAM-A normalized to actin expression in SK-BR-3 after (**a**) JAM-A knockdown and (**b**) HER3 knockdown. (**c**) Cell viability measured by MTT assay in control MCF7 cells overexpressing empty vector (MCF7), MCF7 cells overexpressing JAM-A (MCF7-JAM+) and SKBR-3 breast cancer cell lines after 72h of knockdown showing reduction in proliferation in all cell lines after JAM-A or HER3 knockdown. (**d**) Colony formation assay at day 9 revealing that JAM-A and HER3 knockdown completely inhibited colony formation of MCF7 and SKBR3 cells compared to siNEG control, while HER2 knockdown only affected colony formation in HER2-positive SKBR3 cells. Experiments were performed three times and data represent mean ± s.e.m, compared using two-tailed, equal variance Student’s *t*-tests, * *p* < 0.05, ** *p* < 0.01, *** *p* < 0.001.

To determine the signaling consequences of expressional alterations in JAM-A and HER3, we focused upon the HER family downstream effectors AKT and ERK as indicators of the PI3K and MAPK pathways (respectively). Gene silencing of either JAM-A (Figure 2a) or HER3 (Figure 2b) significantly reduced the protein levels of pAKT and pERK relative to total AKT/ERK. This was accompanied by cellular viability deficits in MCF7, MCF7-JAM+ and SK-BR-3 breast cancer cells (Figure 2c; *p* < 0.01, *p* < 0.01 and *p* < 0.001 respectively for JAM-A silencing; *p* < 0.001, *p* < 0.01 and *p* < 0.001 respectively for HER3 silencing). As JAM-A-overexpressing cells represented a reductionist model for aggressive JAM-A-overexpressing tumors, it was encouraging that interference with JAM-A expression in this setting was capable of inducing significant functional effects. Quantitatively-similar viability deficits induced by silencing of either gene in HER2-negative (MCF7) and –positive (SK-BR-3) cell lines suggested that JAM-A and HER3 may be in a linear pathway, and that HER3 can regulate cell survival independently of HER2. Gene silencing of either JAM-A or HER3 also reduced colony formation in MCF7 and SKBR3 cells (Figure 2d); while (in control experiments) silencing of HER2 had little effect on colony formation in HER2-negative MCF7 cells but obliterated colony formation in HER2-positive SKBR3 cells (Figure 2D). Successful silencing of HER2 in SKBR3 cells was confirmed in Figure S6.

With evidence that JAM-A regulates HER3 expression at gene expression level in breast cancer cells (and indeed supportive data of a correlation between JAM-A and HER3 gene expression in several cancer types; Figure S7), we next wished to identify genes co-expressed with both JAM-A and HER3. Accordingly, a dataset of 2342 breast cancer samples from 13 independent gene expression studies was analyzed by means of our previous work using weighted gene co-expression network analysis (WGCNA) [29]. However no candidates were identified. We therefore repeated the analysis to identify genes co-expressed with HER3 only, which generated a list of 37 candidates with statistically-significant correlation coefficients of > or < 0.5 (Table S1). Of that list, the disease-free survival hazard ratio (DFS-HR) for 8 gene candidates was also statistically significant (shaded rows; *p* values in final column). Based on an analysis of the literature we selected three candidates known either as transcription factors or to be influenced by JAM-A expression (TJP3, GATA-3 and FOXA1; of which FOXA1 was the only one with a statistically significant link to DFS-HR). TJP3 was subsequently excluded because its mRNA expression was undetectable in the breast cell lines tested (R.G.B. Cruz; personal communication), whilst GATA-3 was excluded since its gene or protein expression were unaffected by alterations in JAM-A expression (Figure S8). Therefore FOXA1 (Hepatocyte nuclear factor 3a; HNF3a), a member of the Forkhead box (Fox) family of transcription factors that reportedly acts as a transcriptional regulator of HER3 in breast cancer cell lines [30,31] was selected for further study.

### 3.3. JAM-A Expression Influences mRNA and Protein Expression of FOXA1

**Figure 3:**
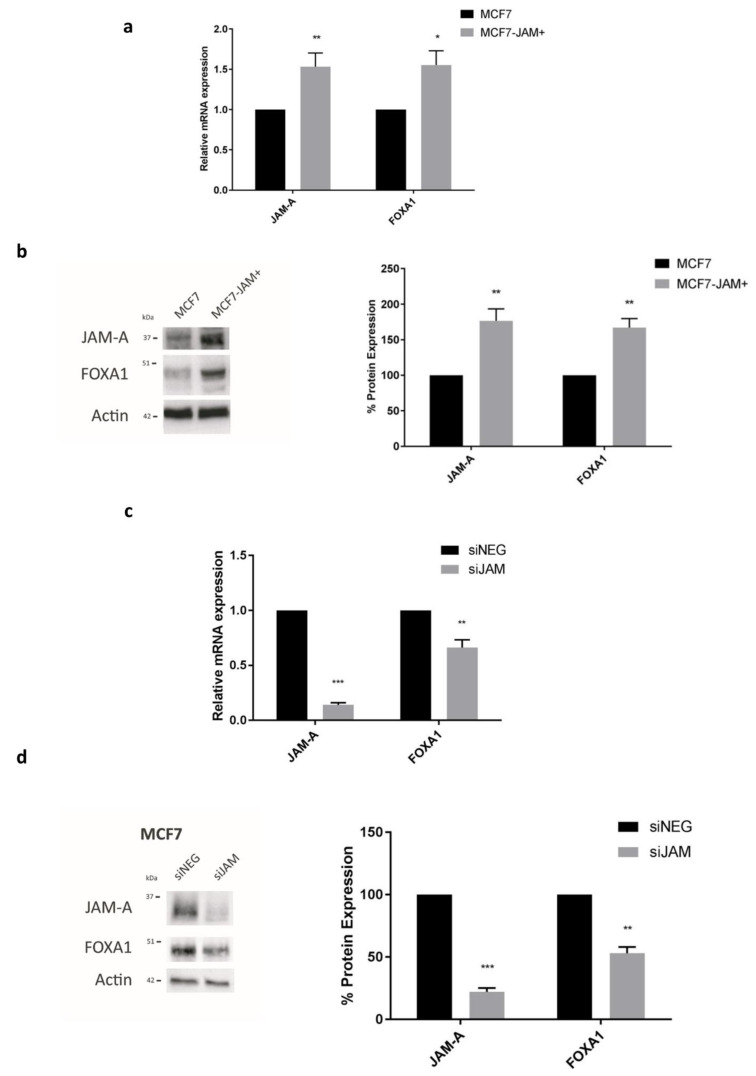
JAM-A influences mRNA and protein expression of FOXA1. RNA and protein from control MCF7 cells overexpressing empty vector (MCF7) and MCF7 cells overexpressing JAM-A (MCF7-JAM+); or MCF7 cells transfected with 25 nM control siRNA (siNEG) or a pool of JAM-A siRNA (siJAM) for 72 h were analyzed by qRT-PCR and immunoblotting. (**a**) Analysis of MCF7-JAM+ by RT-qPCR revealed significant upregulation of FOXA1 mRNA expression compared to that in wild type MCF7 cells. (**b**) Representative western blot image and densitometric analysis of JAM-A and FOXA1 normalized to actin revealed that overexpression of JAM-A in MCF7-JAM+ cells was associated with upregulation of FOXA1 protein expression compared to wild type MCF7 cells. (**c**) RT-qPCR revealed FOXA1 mRNA expression was significantly reduced after JAM-A knockdown compared to control conditions in MCF7 cells. (**d**) Representative western blot image and densitometric analysis of JAM-A and FOXA1 normalized to actin revealed that JAM-A gene silencing reduced FOXA1 protein expression compared to siNEG control in MCF7 cells. Experiments were performed three times and data represent mean ± s.e.m, compared using two-tailed, equal variance Student’s *t*-tests, * *p* < 0.05, ** *p* < 0.01, *** *p* < 0.001.

To test putative links between JAM-A, FOXA1 and HER3, we first examined whether FOXA1 expression levels in breast cancer cells were sensitive to manipulation of JAM-A levels. JAM-A overexpression significantly increased FOXA1 mRNA (Figure 3a) and protein expression (Figure 3b) in MCF7 cells compared to empty vector-MCF7 cells. JAM-A gene silencing also significantly reduced the mRNA (Figure 3c) and protein (Figure 3d) expression of FOXA1 in MCF7 cells. These findings were replicated in SK-BR-3 cells (Figure S9).

### 3.4. FOXA1 Regulates HER3 mRNA and Protein Expression

**Figure 4:**
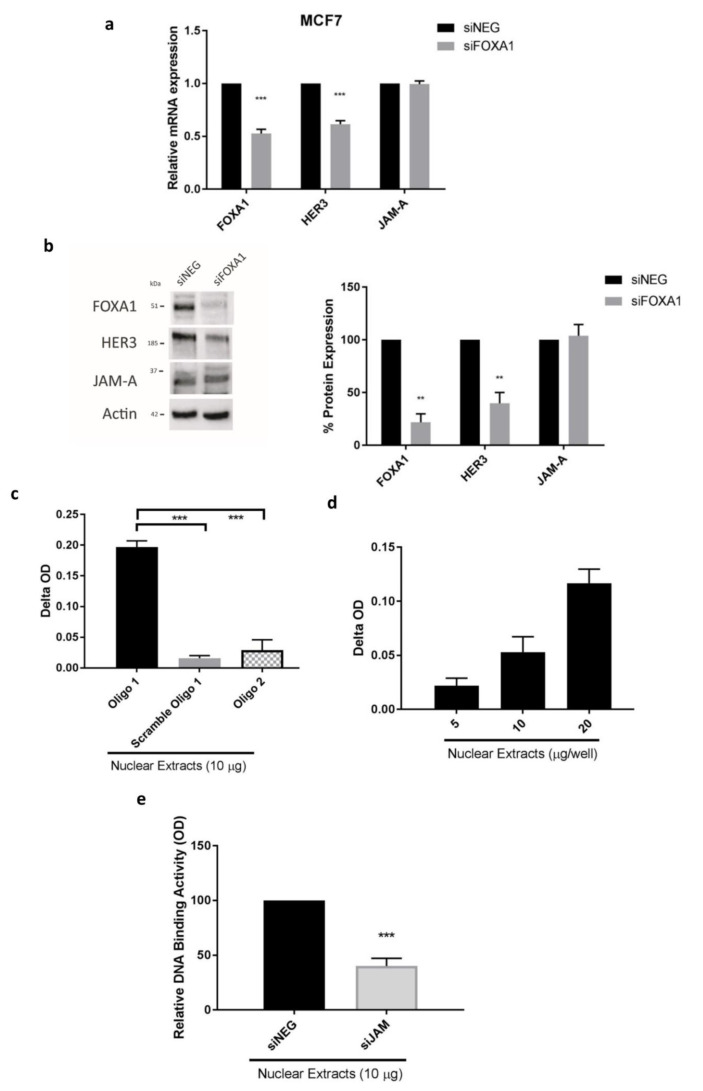
FOXA1 is a putative transcription factor controlling the mRNA and protein expression of HER3. RNA and protein from MCF7 cells transfected with 25 nM control siRNA (siNEG) or a pool of siRNA targeting FOXA1 for 72 h were extracted for analysis. (**a**) qRT-PCR revealed that HER3 mRNA expression was reduced after FOXA1 gene silencing, while JAM-A mRNA expression was not affected; (**b**) Representative western blot image and densitometric analysis for HER3 and JAM-A protein normalized to actin expression after FOXA1 knockdown revealed that HER3 protein expression was affected by FOXA1 knockdown, while it had no effect on JAM-A expression. Nuclear proteins of untreated MCF7 cells were extracted and subjected to protein-DNA binding assays using specific oligonucleotides that represented putative FOXA1 binding sites in the HER3 gene promoter. (**c**) Results of the colorimetric measurement of the reactions (Binding Activity = delta optical density (sample—blank) × sample dilution) of nuclear protein binding activity to different sequences representing the HER3 gene promoter revealed that FOXA1 had high affinity binding only for the sequence of Oligonucleotide 1. (**d**) Increasing concentrations of nuclear protein elicited proportional increases in optical density values of FOXA1 binding to the oligonucleotide representing a selected region of the HER3 gene promoter. (**e**) Nuclear proteins were extracted from MCF7 cells after 72 h of transfection with JAM-A siRNA and subjected to protein-DNA binding assays using Oligonucleotide 1. Relative optical densities revealed that FOXA1 binding to this representative sequence from the HER3 gene promoter was decreased in JAM-A knockdown compared to control conditions. Experiments were performed three times and data represent mean ± s.e.m, compared using two-tailed, equal variance Student’s *t*-tests, ** *p* < 0.01, *** *p* < 0.001.

Having established JAM-A-dependent regulation of FOXA1 expression, we next tested the capacity of FOXA1 to alter HER3 levels. Transient gene silencing of FOXA1 significantly reduced HER3 mRNA (Figure 4a) and protein (Figure 4b) expression in MCF7 cells; while JAM-A mRNA and protein levels were unaffected by FOXA1 silencing (Figure 4a,b). Results were validated in SK-BR-3 cells (Figure S10).

Having confirmed that FOXA1 regulates HER3 expression, we next used data from the ENCODE (Encyclopedia of DNA Elements) project (https://www.encodeproject.org/ (accessed on 10 May 2020)) to identify FOXA1 transcription factor binding regions in the proximal promoter of HER3 in breast cancer cells. Specifically, using CHIP-seq, the ENCODE project identified two FOXA1 binding regions in the CpG island 5’ to the RefSeq-annotated HER3 transcription start site (TSS) in T-47D breast cancer cells. This identified the binding regions of FOXA1 in the HER3 promoter as a 243bp (chr12:56473387–56473630, hg19 assembly) and a 239bp (chr12:56473753–56473992, hg19 assembly) segment of the CpG island (chr12:56472793–56474393, hg19 assembly) proximal to the HER3 TSS. A visual representation of the promoter region of the HER3 gene is shown in Figure S11, highlighting the two potential FOXA1 binding sites. However, despite localizing the binding region of FOXA1 in the HER3 promoter, the actual binding sites for all transcription factors including FOXA1 are less than ~20 bp in length (a key limitation of the CHIP-seq approach). To further localize the target region of FOXA1, we therefore searched these two promoter segments for putative FOXA1 transcription factor binding sites (TFBS) using FOXA1 TFBS binding site information from TRANSFAC [24] and the tffind search tool from the Piptools package [25].

Protein-DNA binding assays were next used to determine if FOXA1 bound to the identified HER3 promoter sequence in our cell line models (the ENCODE project used T-47D cells). Specifically, oligonucleotide sequences were commercially synthesized to represent the HER3 gene sequences predicted to contain TFBS for FOXA1, forming part of the FOXA1 target regions identified from the ENCODE project. As shown in Figure 4c, we identified one sequence from the HER3 gene (represented by synthetic Oligo 1) that showed high binding to FOXA1 in nuclear extracts from MCF7 cells; compared to the scrambled oligonucleotide control and to another sequence from the HER3 gene promoter also recognized as a putative FOXA1 TFBS (represented by synthetic Oligo 2). Moreover, we observed a proportional increase in FOXA1 binding activity to the HER3 promoter sequence represented by Oligo 1 with increasing concentrations of nuclear protein, indicating that the binding is specific (Figure 4d). The fact that one of the TFBS did not confirm experimentally can be attributed to the fact that the TFBS were predicted using data from TRANSFAC. This is a common problem with TFBS predictions, as the experimentally-determined binding sites upon which these searches are based are short and degenerate. 

Since evidence thus far suggested a pathway whereby JAM-A regulates the expression of FOXA1 which in turn binds to putative FOXA1 binding sequences within the HER3 gene promoter and upregulates HER3 transcription, we wanted to verify that JAM-A silencing, by reducing FOXA1 expression, would reduce interactions between FOXA1 and its putative HER3 promoter binding site (represented by Oligo 1). JAM-A silencing indeed reduced the binding reaction between Oligo 1 and FOXA1 from MCF7 nuclear extracts (Figure 4e; *p* < 0.001), presumably reflecting lower levels of FOXA1 secondary to JAM-A silencing. While chromatin immunoprecipitation and in vitro transcription assays would ultimately be required for final experimental proof, our results nonetheless suggest a novel model whereby JAM-A regulates the expression of HER3 in breast cancer cells (independently of HER2 expressional status) by first altering the expression of the known HER3 transcription factor FOXA1. That this represents a uni-directional pathway is supported by Figure S12, with evidence that HER3 gene silencing did not affect either JAM-A or FOXA1 gene/protein expression.

### 3.5. JAM-A Influences FOXA1 Transcription by Altering β-Catenin Expression and Localization

**Figure 5:**
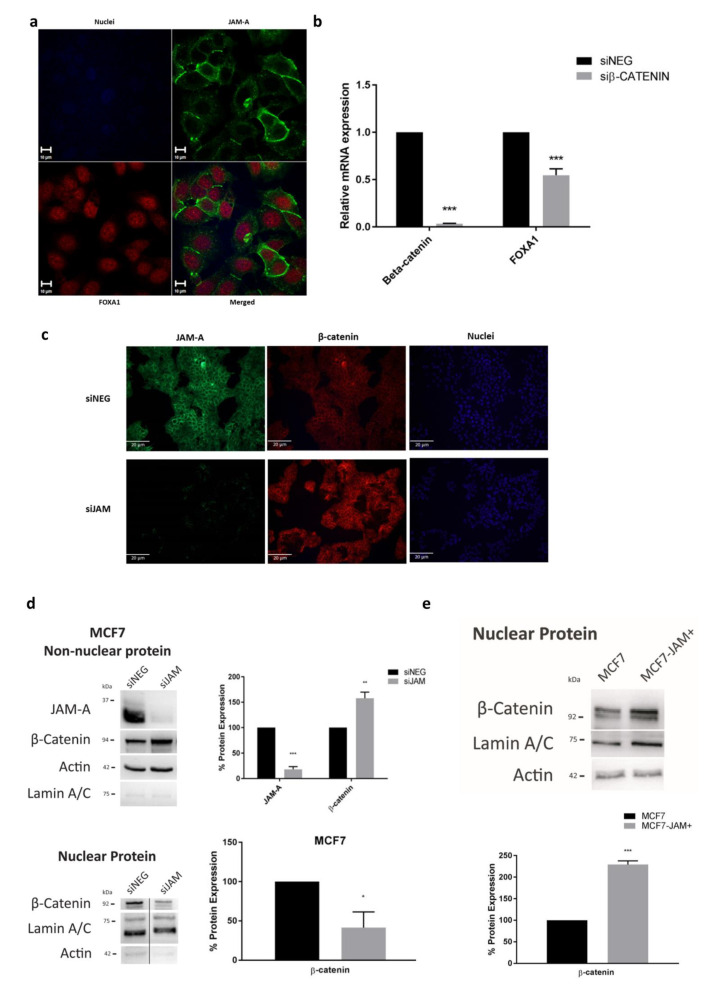
JAM-A expression influences β-catenin presence in the nuclear and non-nuclear fraction of breast cancer cells. (**a**) MCF7 cells were subjected to double immunofluorescence labeling for JAM-A and FOXA1, with a counter-stain of DAPI (blue) to highlight the nuclei. Confocal microscopy for JAM-A (green) and FOXA1 (red) revealed no spatial overlap of these proteins within the cells. (**b**) MCF7 cells were transfected with 25 nM of control siRNA (siNEG) or a pool of β-catenin siRNA and harvested 72 h later for qRT-PCR analysis. Results show FOXA1 mRNA expression was reduced following knockdown of β-catenin. (**c**) Immunofluorescence labelling of MCF7 cells transfected with 25 nM of control siRNA (siNEG) or a pool of JAM-A siRNA (siJAM) for 72 h revealed that JAM-A (green) knockdown increased the membranous and cytoplasmic intensity of β-catenin (red). (**d**) Upregulation of β-catenin in non-nuclear fractions of MCF7 cells after JAM-A knockdown was confirmed by western blot analysis (normalized to actin). β-catenin recovery from nuclear fractions of MCF7 cells was reduced after JAM-A knockdown (normalized to Lamin A/C). (**e**) Representative western blot image and densitometric analysis of nuclear β-catenin protein expression normalized to Lamin A/C revealed increased β-catenin in MCF7 cells overexpressing JAM-A (MCF7-JAM+) relative to cells overexpressing empty vector MCF7). Experiments were performed three times and data represent mean ± s.e.m, compared using two-tailed, equal variance Student’s *t*-tests, * *p* < 0.05, ** *p* < 0.01, *** *p* < 0.001.

To begin investigating mechanistic links between JAM-A and FOXA1, we analyzed the localization of both proteins in MCF7 cells by confocal immunofluorescence microscopy. Since JAM-A staining was mostly membranous while FOXA1 localized in the nuclei (Figure 5a), it was considered unlikely that JAM-A-dependent regulation of FOXA1 involved their direct physical contact. As SOX17 associated with β-catenin has been shown to regulate the transcription of FOXA1 in endodermal and colorectal cancer cells [32], and β-catenin is reportedly influenced by JAM-A expression [33], we investigated whether JAM-A expressional alterations would change SOX17 expression. As JAM-A overexpression did not alter SOX17 levels in nuclear extracts of either MCF7 or SK-BR-3 cells (Figure S13), we focused directly on β-catenin.

First, gene silencing of β-catenin was confirmed to significantly reduce FOXA1 gene expression in MCF7 (Figure 5b) and SK-BR-3 cells (R.G.B. Cruz; personal communication). We next sought to investigate whether this could occur downstream of JAM-A silencing. Surprisingly, JAM-A silencing visually increased β-catenin expression in MCF7 cells (Figure 5c); but this was mostly localized in non-nuclear compartments where it would be incapable of activating the FOXA1 gene promoter. Subcellular fractionations in MCF7 cells confirmed these results on a biochemical level, with JAM-A silencing increasing recovery of non-nuclear β-catenin (Figure 5d; *p* < 0.01) and reducing recovery of nuclear β-catenin (Figure 5d, *p* < 0.05). These results were replicated in SK-BR-3 cells (Figure S14). Correspondingly, overexpression of JAM-A increased nuclear β-catenin recovery in MCF7 (Figure 5e; *p* < 0.001) and SK-BR-3 (Figure S15) cells. 

Unexpectedly, JAM-A overexpression also induced smaller but significant increases in β-catenin recovery from non-nuclear fractions in MCF7-JAM+ and SK-BR-3-JAM+ cells (Figure S15). Although longer-term investigations would be necessary to understand why, it is possible that JAM-A influences β-catenin expression/localization in different ways. Specifically, in the gene silencing scenario, β-catenin recruitment to the cell membrane may be required to compensate for compromised cell adhesion/barrier function downstream of JAM-A loss. In the overexpression scenario, the ability of JAM-A to increase total expression of β-catenin likely just dictates that more protein is available in both non-nuclear and nuclear compartments (without necessarily having a stoichiometric binding relationship at the cell membrane). Either way, the overall findings support our hypothesis that nuclear β-catenin abundance is affected by JAM-A expression, which in turn would influence FOXA1 transcription and drive changes in HER3 expression. 

### 3.6. JAM-A Expression Correlates with Proliferation in a Semi-In Vivo Setting and with β-Catenin Expression in a Patient Tissue Microarray of Invasive Breast Cancer Specimens

We next sought to test our hypothesis in more translational models. Chick embryo xenograft models have been widely used to study angiogenesis, tumor invasion and metastasis [34,35,36,37], so we tested the effect of JAM-A silencing or overexpression on human breast tumor xenografts. The use of siRNA-transfected cells in this model has previously been validated in studies on human glioblastoma [38] and breast cancer [39]. Accordingly, MCF7 cells were implanted onto the chorioallantoic membrane of 15 eggs and a solution containing control siNEG (*n* = 7) or JAM-A siRNA (*n* = 8) applied directly on top. After 6 days of incubation, xenografts were excised and prepared for immunohistochemistry. 

**Figure 6:**
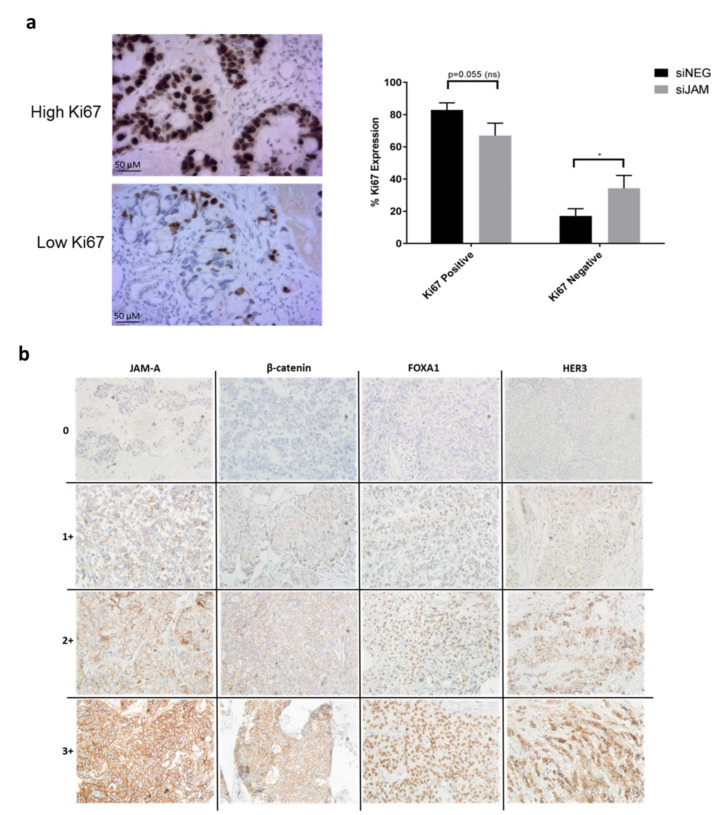
JAM-A is associated with tumor cell proliferation and other oncogenes in higher order models. (**a**) Images—representative high and low Ki67 staining from different tumor xenograft samples. Graph—Quantification of Ki67 in 500 cells revealed that JAM-A silencing was associated with a higher number of Ki67-negative cells, resting cells, compared to control conditions. Data represent mean ± s.e.m, compared using one-tailed, equal variance Student’s *t*-tests, * *p* < 0.05. (**b**) Representative images at 10X magnification illustrating 0, 1+, 2+ and 3+ JAM-A, β-catenin, FOXA1 and HER3 scoring intensities in a tissue microarray constructed from invasive breast cancer patient specimens. JAM-A expression was predominantly membranous with some cytosolic expression. β-catenin was mostly present in the cytoplasm and/or membrane but occasionally in the nucleus. FOXA1 expression was predominantly nuclear. HER3 expression was both membranous and cytoplasmic.

While the efficacy of JAM-A gene silencing in situ on chick embryo xenografts was disappointingly low relative to that which would have been achieved in vitro, there was however mild focal loss of JAM-A expression affecting up to three small groups of cells in the same area in 4/8 JAM-A-silenced tumors but 0/7 control tumors examined (Figure S16). In these focal areas of JAM-A loss, corresponding losses of β-catenin and HER3 (Figure S16) but not FOXA1 (H. Jahns; personal communication) were observed. Furthermore (Figure 6a), assessment of cell proliferation (via enumeration of Ki67-positive and -negative cells in 500 tumor cells) revealed a trend towards more Ki67-positive cells in control xenografts relative to JAM-A-silenced xenografts (*p* = 0.055). The latter had significantly more Ki67-negative cells than controls (Figure 6a; *p* < 0.05 by one-tailed equal variance unpaired Student’s *t*-tests). This result indicates that JAM-A knockdown is associated with reduced proliferation within the tumor cells, and is consistent with in vitro data presented earlier. This is particularly encouraging given the very low efficacy of JAM-A silencing in situ.

When JAM-A-overexpressing cells were utilized in the chick embryo xenograft model, small pockets of intense JAM-A cytoplasmic staining were observed on these xenograft tumors but not in wild type controls examined (Figure S17). Cytoplasmic JAM-A would not ordinarily be expected in normal polarized breast epithelial cells, but its presence is unremarkable in the context of JAM-A-overexpressing cells that were generated to model aggressive and unpolarized JAM-A-overexpressing breast tumors. Labeling for HER3 and β-catenin was subtly of higher intensity in 4/4 instances of JAM-A-overexpressing xenografts examined, but not in wild type controls (Figure S17). 

Finally we analyzed JAM-A expression in a tissue microarray (TMA) composed of cores from 144 patients with invasive breast cancer. Details of clinical and pathological characteristics for each patient are depicted in Table S2. JAM-A was scored using a system of 0 (negative), 1+, 2+ or 3+ according to the intensity of membranous JAM-A staining in tumor cells (Figure 6b). JAM-A was stratified as high (2+ and 3+) or low (0 and 1+) and results were compared with clinicopathological parameters (Table 1). Results revealed that JAM-A expression by itself was not statistically associated with age or HER2 expression in the population studied. It is important to note that the population numbers were strongly biased in favor of older patients (>50 years) and those who were HER2-negative. Since we had already reported statistically significant correlations between JAM-A expression and both lower patient age and HER2 positivity in larger patient cohorts [13], this reverse bias in the current small patient population would have influenced the analysis. Regardless, high JAM-A expression did significantly correlate with high grade tumors (*p* = 0.017), triple-negative and Luminal B molecular subtypes (*p* = 0.048), ER-negativity (*p* = 0.019) and PR-negativity (*p* = 0.004). The TMA was also stained for other proteins of interest in the pathway described before. Similarly to JAM-A expression, β-catenin, FOXA1 and HER3 expression were stratified as high (2+ and 3+) or low (0 and 1+) (Figure 6B). 

β-Catenin was also analyzed according to its absence (score = 0) or presence in each of the cellular compartments. The localization was used to determine its putative activation status as a transcription factor, with membranous staining suggesting inactive and cytosolic/nuclear staining suggesting active protein [40,41]. A detailed analysis of the frequencies of the parameters observed in the TMA is presented in Table S3. We then examined the relationship between high versus low expression of JAM-A and the other proteins of interest. High JAM-A expression was significantly associated with high β-catenin expression (*p* = 0.003), but not with high FOXA1 or HER3 expression (Table 2, Table S4). The fact that high JAM-A expression was unexpectedly not associated with high expression of FOXA1 and HER3 likely reflects the limited size and molecular subtype distribution of this TMA. For example, coincident high expression of JAM-A, FOXA1 and HER3 was observed in the Luminal B subtype, but it was unfortunately not possible to further investigate their association due to the small number of Luminal B patients in which these proteins were scoreable (*n* = 8) (Table S2). High JAM-A expression was more associated with the small number of triple-negative breast cancer cases; high FOXA1 and HER3 were highly associated with the Luminal A subtype; and high β-catenin was not significantly correlated with any one molecular subtype. 

As the small size and relative lack of molecular subtype diversity in our patient population hampered direct correlations between JAM-A expression and FOXA1/HER3, we instead elected to analyze the expression levels of each protein in the context of its next downstream protein in our proposed pathway. As shown in Table 2, high JAM-A expression was associated with high β-catenin expression (*p* = 0.003) but not β-catenin subcellular localization. In turn, high β-catenin expression correlated significantly with high FOXA1 expression (*p* = 0021); and high FOXA1 expression correlated significantly with high HER3 expression (*p* = 0.000). Taken together, our findings are consistent with a linear model in which high expression of JAM-A influences nuclear β-catenin levels, in turn increasing FOXA1 expression which then promotes HER3 expression in breast cancer settings.

## 4. Discussion

In the context of breast cancer, overexpression of HER3 has been reported in approximately 18% of patients, and is associated with poor prognosis and reduced survival [5]. However estimates vary, with one study reporting overexpression of HER3 in 67% of comedo ductal carcinoma in situ (DCIS), 52% of invasive ductal carcinomas, 71% of mixed in situ/invasive carcinomas and 25% of invasive lobular carcinomas [42]. Considering the genomic subtypes of breast cancer, HER3 expression has been linked with poor disease-free survival in both HER2 and triple-negative subtypes, in addition to poorer overall survival in the latter. HER3 overexpression has also been reported as an important prognostic marker in hormone receptor-negative breast cancer [43]. 

Besides its high expression and association with the development of breast cancer, compensatory HER3 expression has also been implicated in the development of resistance to anti-HER2 targeted therapies [6,7]. It is therefore timely and important to investigate factors driving its expression or function in cancers including that of the breast. We previously reported a link between resistance to HER2-targeted therapies in breast cancer patients and a protein called JAM-A [20], itself a putative regulator of HER2 expression [13]. High JAM-A expression predicts early tumor recurrence and reduced life expectancy in breast cancer patients [11,13,19]; likely via its influence on regulatory processes like cell migration, proliferation and apoptosis [12,17,19]. In this study we provide the first evidence of JAM-A acting as an upstream regulator of HER3, which may have high relevance to the development of resistance to HER2-targeted therapies. 

Specifically, we showed that overexpression of JAM-A upregulated HER3 mRNA and protein levels, whilst gene silencing of JAM-A reduced HER3 mRNA and protein expression. Although our previous publication described JAM-A-dependent regulation of HER2 to be at a translational/post-translational level [13], this manuscript provides the first evidence that JAM-A-dependent regulation of HER3 expression can also be at a transcriptional level (whether sequential or concomitant is unknown at present). This is consistent with existing knowledge that, in contrast to HER2, HER3 overexpression is more likely to result from increased gene transcription than gene amplification [9].

To identify potential mechanisms of crosstalk between JAM-A and HER3, we investigated genes co-expressed with HER3 using weighted gene coexpression network analysis (WGCNA). This method comprises a powerful ‘guilt-by-association’-based method to extract co-expressed groups of genes from large heterogeneous messenger RNA expression data sets [29], and allowed us to select potential transcription factors capable of regulating HER3 transcription and being regulated by JAM-A expression. Of greatest interest was FOXA1. It has been reported that FOXA1 is overexpressed in prostate [44], esophageal and lung [45] cancer. In breast cancer, its overexpression has been shown to correlate with specific subtypes such as molecular apocrine [46] and luminal A [47], and breast cancer metastases that are resistant to endocrine therapy [48]. Collectively this suggests that FOXA1 may significantly contribute to pro-tumorigenic phenotypes [49].

FOXA1 has also been described as a key transcription factor that binds to the promoters of more than 100 genes, which in turn regulate many cellular functions [50]. One of the known targets of FOXA1 is HER3 [30,31]. Studies have shown that FOXA1 predominantly binds to enhancer regions distal to transcriptional start sites [51,52]. Other studies based upon ChIP-Seq and ChIP-PCR in breast cancer cells have indicated that FOXA1 binds to the HER3 gene [30,31]. Moreover, one study showed that FOXA1 knockdown reduced HER3 gene expression in a panel of breast cancer cell lines [31], corroborating our results showing that FOXA1 gene silencing reduced HER3 protein expression. 

In our hands JAM-A gene silencing significantly reduced FOXA1 levels, suggesting that JAM-A-dependent regulation of HER3 expression may occur through FOXA1. In contrast, FOXA1 or HER3 silencing had no effect on JAM-A expression, suggesting that JAM-A acts upstream of both FOXA1 and HER3 and regulates their expression in a unidirectional manner. Based on our results suggesting that JAM-A regulates the expression of FOXA1 at a transcriptional level, we investigated mechanisms by which this process might happen. Our results showing the localization of JAM-A and FOXA1 in different subcellular compartments would preclude the likelihood of a direct physical interaction. In this context, the existence of an intermediate player connecting both proteins was considered. 

Our investigation focused initially on SOX17, since it in association with β-catenin has been reported to regulate FOXA1 transcription [32]. However, manipulation of JAM-A expression did not affect nuclear levels of SOX17. We next focused directly on β-catenin, since it has been reported as necessary for SOX17 transcription of FOXA1 [32] and associated with regulating the transcription factor ZEB-1 by the tight junction protein Claudin-1 [53] in colon cancer cells.

β-catenin gene silencing significantly reduced FOXA1 gene expression in breast cancer cells. As β-catenin translocation to the nucleus is a necessary step to initiate transcription [54], we also confirmed β-catenin localization in the nuclear fractions of breast cancer cells and a relationship between JAM-A expression and nuclear β-catenin levels. Interestingly, both JAM-A overexpression and knockdown also increased β-catenin expression in non-nuclear fractions; a finding we speculate associates with compensatory functions of β-catenin in cellular adhesion [55,56] rather than tumorigenesis.

We next tested whether JAM-A expression would influence breast cancer proliferation using an in ovo/semi-in vivo approach by performing JAM-A gene silencing in cells implanted on the chick embryo chorioallantoic membrane (CAM), as previously described [39]. Our results revealed that, although levels of JAM-A were not consistently reduced, this was sufficient to alter levels of the proliferation marker Ki67 in cells treated with JAM-A siRNA and to produce some focal losses in HER3 and β-catenin. Of note, we performed the siRNA transfections in situ in an attempt to mimic a more realistic tumor microenvironment, where xenograft tumors would have started to develop in situ prior to the application of any JAM-A-targeted intervention. However, in future studies it is likely that pre-silencing JAM-A prior to implantation as well as once again during xenograft growth may yield more fruitful results. It would also be interesting to test other parameters such as invasion in this context using different cell lines, since MCF7 cells have been reported not to be invasive in vitro [57,58].

We then adopted a translational approach in seeking to confirm in vitro results from breast cancer cell lines with experimental data from a tissue microarray (TMA) of patients with invasive breast cancer. High expression of JAM-A has previously been associated with high tumor grade, larger tumor size, lower patient age, low PR levels, ER negativity, HER2 positivity, and Luminal B, HER2+ and basal subtypes of breast cancer [11,13]. Individually and collectively, these findings spell poor prognosis for patients. In accordance with those previous studies, our analysis confirmed that high JAM-A is associated with poor prognostic markers like high tumor grade, larger tumor size; ER and PR negativity; Luminal B and triple negative subtypes. However, JAM-A expression had no statistical association with HER2 expression or age in the tissue from the TMA we tested, reflecting the very small number of patients who had HER2-positive tumors or were younger than 50 years old; further evidencing the inequal distribution amongst breast cancer subtypes and the late incidence within the population tested. 

As our study had led us to a linear mechanism connecting JAM-A, β-catenin, FOXA1 and HER3 (in that order), we analyzed expressional associations between those proteins in the 144-patient breast cancer TMA. While the TMA size and heterogeneity was under-powered to detect direct correlations between high expression of JAM-A and HER3, it nonetheless confirmed statistically significant associations between each protein and its downstream neighbor in our proposed pathway. Specifically, high JAM-A expression correlated with that of β-catenin, high expression of β-catenin correlated with that of FOXA1, and high expression of FOXA1 correlated with that of HER3. It must be noted that an important characteristic within our TMA patient population was the high incidence of tumors classified as Luminal A (ER+/PR+/HER2-)—almost 80%. Although β-catenin and HER3 expression have not been shown to associate with hormone receptor status [43,59], JAM-A expression has been correlated with negative ER/PR expression [13], while FOXA1 correlates with ER-positivity [60]. This could account for unexpected correlations within our TMA, which would have factored less in larger patient populations or those with greater heterogeneity in breast cancer molecular subtype.

While comprehensive future investigations will be needed to elucidate the elusive link between β-catenin and JAM-A, the Hippo tumor suppressor protein paralogues Yes-associated protein (YAP) and WW-domain-containing transcription regulator 1 (WWTR1; TAZ) may represent a potential starting point. Specifically, it has previously been shown that concurrent loss of YAP/TAZ (representing active Hippo signaling) reduces β-catenin expression and attenuates pro-tumorigenic signaling via (amongst others) inhibition of the PI3K pathway [61]. Our preliminary data showed a trend towards increased YAP/TAZ protein expression in JAM-A overexpressing breast cancer cells (Figure S18); potentially suggesting a mechanism to account for increased β-catenin expression and transcriptional activity in the JAM-overexpressing tumorigenic cellular model. However it has alternatively been suggested that YAP/TAZ binds β-catenin and prevents its nuclear translocation, exerting a negative tone on its transcription factor activities [62]. Accordingly, the complexities of Hippo-dependent versus –independent regulation of β-catenin in breast cancer settings require comprehensive separate investigations. It is nonetheless intriguing to mention our preliminary evidence that deletion of the PDZ-binding domain of JAM-A increases nuclear accumulation of β-catenin and reduces its non-nuclear levels (R.G.B. Cruz; personal communication). JAM-A can exist in a complex with ZO-1, ZO-2 and YAP/TAZ [63,64], and our preliminary data suggest that JAM-A loss downregulates ZO-1 and ZO-2 (C.E. Richards; personal communication) but not YAP/TAZ (Figure S18). Future studies would therefore merit testing if upregulation of JAM-A in our tumorigenic model would increase the levels of a complex containing JAM-A, ZO-1, ZO-2 and YAP/TAZ. Since loss of YAP/TAZ has been linked with reduced β-catenin expression and reduced pro-tumorigenic signaling [61], the corollary would suggest that upregulation of YAP/TAZ would increase β-catenin expression and potentially tumorigenic signaling. The possibility that β-catenin acts thusly to regulate FOXA1 expression (and subsequently that of HER3) requires a more in-depth investigation beyond the scope of this study.

Returning to the potential clinical significance of this pathway, JAM-A-dependent regulation of FOXA1 is also important because of the latter’s connection to endocrine resistance. FOXA1 expression has been shown to positively associate with ER+ breast cancer [47], and its overexpression has been correlated with endocrine resistance via its capacity to trigger oncogenic gene signatures and pro-resistance proteomic profiles [65]. If pharmacological inhibition of JAM-A, like JAM-A gene silencing, could attenuate FOXA1 levels, it will be exciting in future studies to test whether targeting JAM-A could contribute to overcoming endocrine resistance by acting as an upstream suppressor of FOXA1 expression in endocrine-resistant breast cancers. 

Like the involvement of FOXA1 in endocrine resistance, compensatory HER3 expression has also been reported to play an important role in resistance to anti-HER2 therapies. Co-expression of HER2 and HER3 has been commonly reported as a sign of poor patient prognosis [8]. Moreover, HER3 upregulation after HER2 inhibition has been shown to re-establish tumorigenic signaling via the PI3K pathway [6]. It is currently unknown whether JAM-A can directly drive HER3 upregulation during the acquisition of resistance to HER2-targeted therapies, either in patients or in drug-resistant cell line models; and this will be important to test in future studies. However upregulation of HER3 following the acquisition of resistance to the HER2-targeted drug trastuzumab has been shown in a number of breast cell lines [66], and our own preliminary data suggest that HER3 is upregulated at both gene and protein level in SK-BR-3 cells that have become resistant to trastuzumab or lapatinib (A.O. Leech; personal communication). That HER3 upregulation in drug-resistant SK-BR-3 cells occurs alongside the regulated elaboration of high levels of cleaved JAM-A [20] may be no coincidence, and is particularly intriguing in light of evidence that cleaved JAM-A has biological activity which could promote functional behaviors associated with tumorigenesis [20] [67]. Taken together, since direct anti-HER3 therapeutic options are limited (because of its lack of a tyrosine kinase domain), we suggest that JAM-A represents a novel target meriting investigation to reduce compensatory HER3 expression/signaling in cancer patients resistant to HER2-targeted therapies.

## 5. Conclusions

In conclusion, this manuscript describes a novel pathway involving JAM-A-dependent expressional regulation of HER3 in breast cancer settings by modulating first β-catenin localization and subsequently FOXA1 expression. Given that HER3 upregulation is an important mechanism of acquired resistance to HER2-targeted therapies in breast cancer patients, we suggest that JAM-A antagonists merit future investigation for potential bioactivity in overcoming resistance to HER2-targeted therapies.

## Figures and Tables

**Table 1:** Correlation between JAM-A high versus low protein expression with clinicopathological parameters in invasive breast cancer TMA. Individual parameters and their corresponding total patient numbers are noted in bold type.

Parameter	*n*	High JAM-A	%	Low JAM-A	%	*p*-Value
**Age (years)**	**126**					0.397
≤50	18	9	16.1	9	12.9	
>50	108	47	83.9	61	87.1	
**Tumor Size (mm)**	**126**					0.035 *
≤20	62	22	39.3	40	57.1	
>20	64	34	60.7	30	42.9	
**NHG**	**125**					0.015 *
G1	16	2	3.6	14	20.0	
G2	56	25	46.4	31	44.3	
G3	53	28	50.0	25	35.7	
**Molecular Subtype**	**120**					0.044 *
Luminal A	95	37	69.8	58	86.6	
Luminal B	6	3	5.7	3	4.5	
HER2+	5	2	3.8	3	4.5	
Triple Negative	14	11	20.8	3	4.5	
**ER Status**	**126**					0.010 *
Negative	18	13	23.2	5	7.1	
Positive	108	43	76.8	65	92.9	
**PR Status**	**126**					0.003 *
Negative	39	25	44.6	14	20.0	
Positive	87	31	55.4	56	80.0	
**HER2 Status**	**123**					0.233
Negative	115	49	90.7	66	95.7	
Positive	8	5	9.3	3	4.3	

NHG = Nottingham Histological Grade; ER = Estrogen Receptor; PR = Progesterone Receptor. Percentage indicates values within the JAM-A group. * *p* < 0.05 by two-sided Fisher’s Exact Test or Chi Square Test.

**Table 2:** Linear correlations between high versus low protein expression of JAM-A, β-catenin, FOXA1 and HER3 in breast cancer patient tissues. Studied variables, and their corresponding patient numbers, are shown in bold type. * denotes statistically-significant values.

Variable	*n*	High JAM-A	%	Low JAM-A	%	*p*-Value
**β-catenin Expression**	120					0.003 *
Low		9	16.4	27	41.5	
High		46	83.6	38	58.5	
**β-catenin Localization**	120					0.124
Membrane		4	7	1	1.5	
Cytoplasm/Nucleus		44	80	49	75.4	
Absent		7	13	15	23.1	
**Variable**	***n***	**High β-catenin**	**%**	**Low β-catenin**	**%**	***p*-value**
**FOXA1 Expression**	120					0.021 *
Low		20	5.7	2	23.5	
High		65	94.3	33	76.5	
**Variable**	***n***	**High FOXA1**	**%**	**Low FOXA1**	**%**	***p*-value**
**HER3 Expression**	122					<0.001 *
Low		51	51.5	22	95.7	
High		48	48.5	1	4	

## Data Availability

Data is contained within the article or Supplementary Material.

## Supplementary Materials

The following are available online at https://www.mdpi.com/2072-6694/13/4/871/s1, Document S1: Raw, uncropped blots for all figures; Figure S1: JAM-A silencing with multiple independent siRNA constructs consistently reduces HER3 expression; Figure S2: JAM-A and HER3 overexpression are related to poorer recurrence-free survival in breast cancer patients; Figure S3: JAM-A overexpression increases HER3 protein expression in breast cancer cells; Figure S4: JAM-A gene silencing reduces HER3 mRNA and protein expression; Figure S5: Relative protein expression levels of JAM-A and HER3 in three breast cancer cell line models; Figure S6: HER2 protein expression was successfully reduced by HER2 gene silencing in HER2-positive SK-BR-3 cells; Figure S7: JAM-A and HER3 gene expression are positively correlated in several cancer types; Figure S8: GATA3 mRNA or protein levels are not affected by JAM-A gene silencing; Figure S9: JAM-A levels influence mRNA and protein expression of FOXA1 in SK-BR-3 cells; Figure S10: FOXA1 regulates expression of HER3 but not JAM-A in SK-BR-3 cells; Figure S11: Visual representation of the promoter region of the ERBB3 (HER3) gene; Figure S12: HER3 gene silencing does not affect JAM-A or FOXA1 expression; Figure S13: JAM-A overexpression does not change nuclear SOX17 levels; Figure S14: JAM-A gene silencing reduces nuclear levels and increases non-nuclear levels of β-catenin protein; Figure S15: JAM-A overexpression increases nuclear and non-nuclear β-catenin expression in breast cancer cells; Figure S16: JAM-A silencing tends to reduce HER3 and β-catenin expression in model chick embryo xenografts; Figure S17: JAM-A overexpression tends to increase HER3 and β-catenin expression in model chick embryo xenografts; Figure S18. Putative mechanism of JAM-A-dependent regulation of HER3 expression; Table S1: Genes coexpressed with HER3 in breast cancer; Table S2: Clinicopathological parameters of breast cancer patients for TMA analysis; Table S3: Expression of JAM-A, β-catenin, FOXA1 and HER3 within the breast cancer TMA; Table S4: Association of JAM-A expression with FOXA1 and HER3 expression with TMA features.
