# Supplementary material for: Human Epidermal Growth Factor Receptor-3 Expression Is Regulated at Transcriptional Level in Breast Cancer Settings by Junctional Adhesion Molecule-A via a Pathway Involving Beta-Catenin and FOXA1
Source: Cancers (Basel). 2021 Feb 19;13(4):871. doi: 10.3390/cancers13040871 (PMC7922773; doi:10.3390/cancers13040871)
Supplement: Supplementary file 1 [file cancers-13-00871-s001.zip › SuppDocS1_raw blots_Figs.docx]

**Figure 1**

| **Fig 1b HER3** | **Original** |
| --- | --- |
| 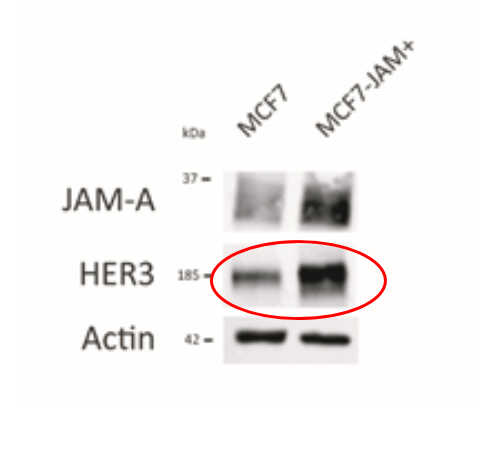 | 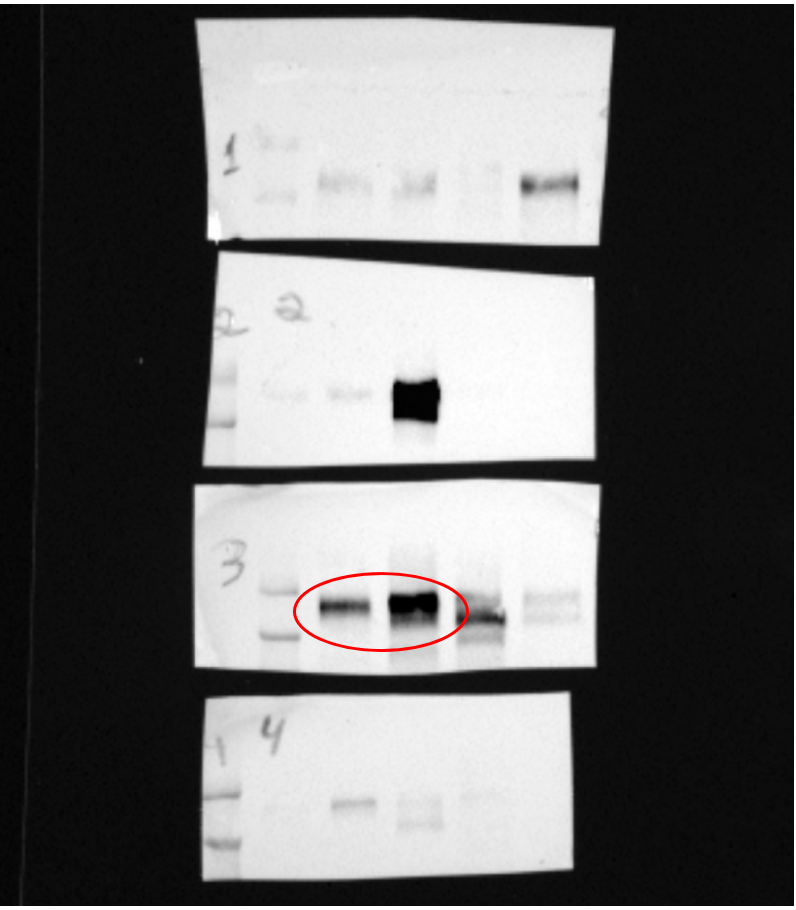  250-  150-  kD: |

| **Fig 1b JAM-A** | **Original**  kD: |
| --- | --- |
| 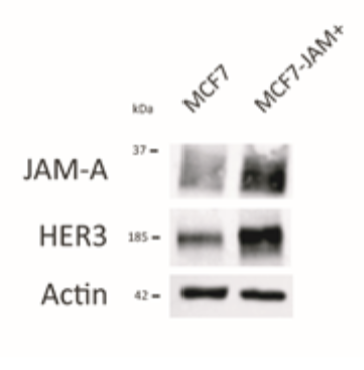 | 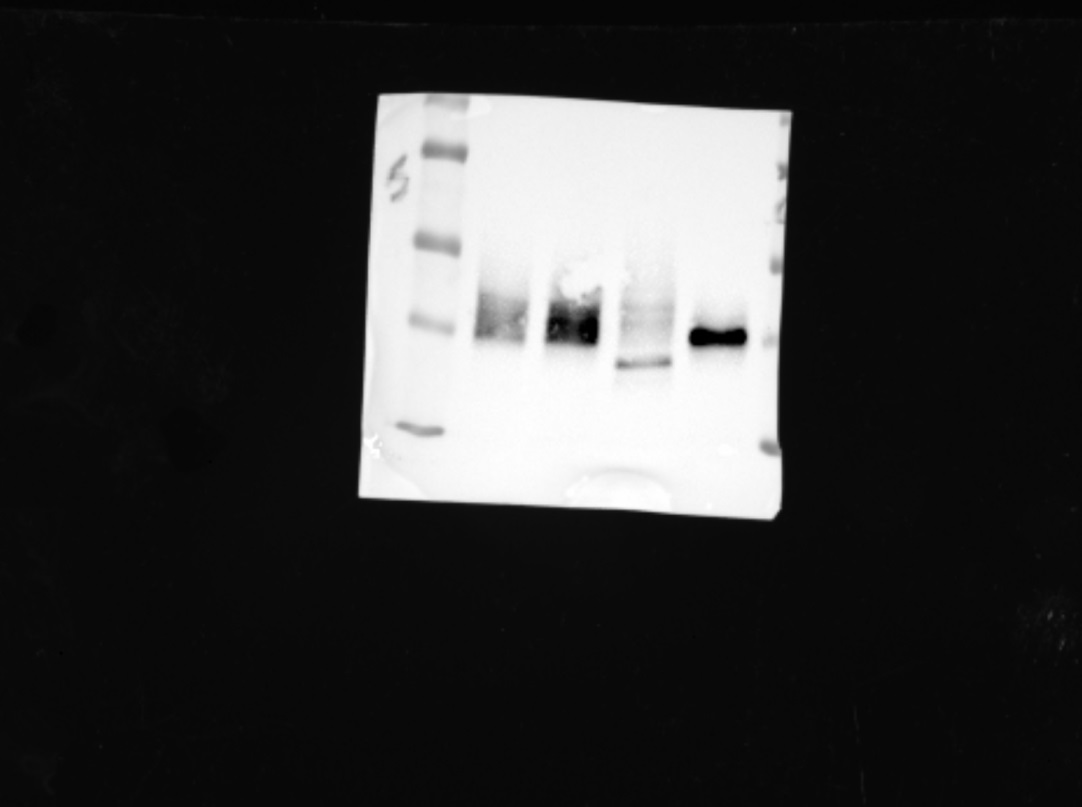  25-  37-  75-  50- |

| **Fig 1b Actin** | **Original**  kD: |
| --- | --- |
| 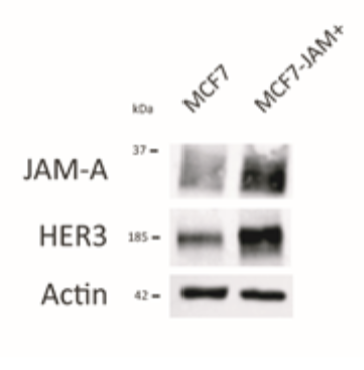 | 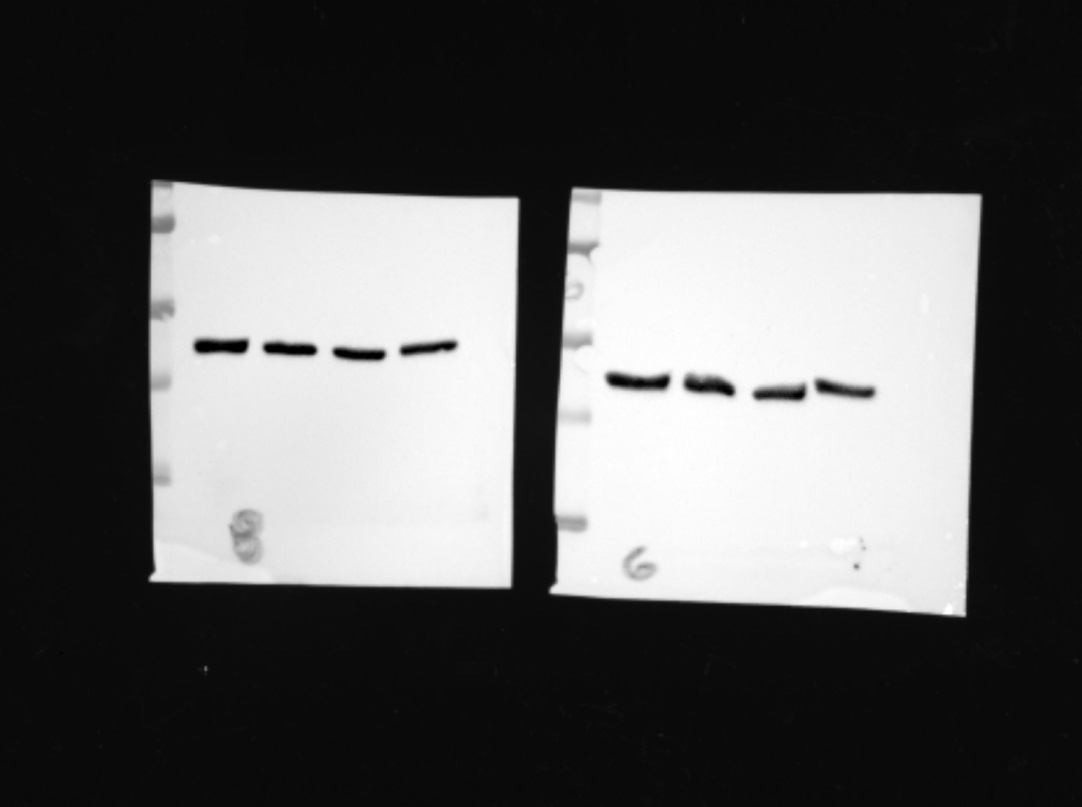  75-  25-  37-  50- |

| **Fig 1d JAM-A** | **Original** |
| --- | --- |
| 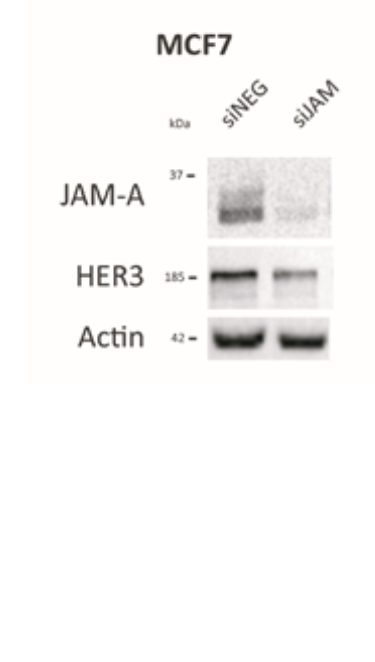 | 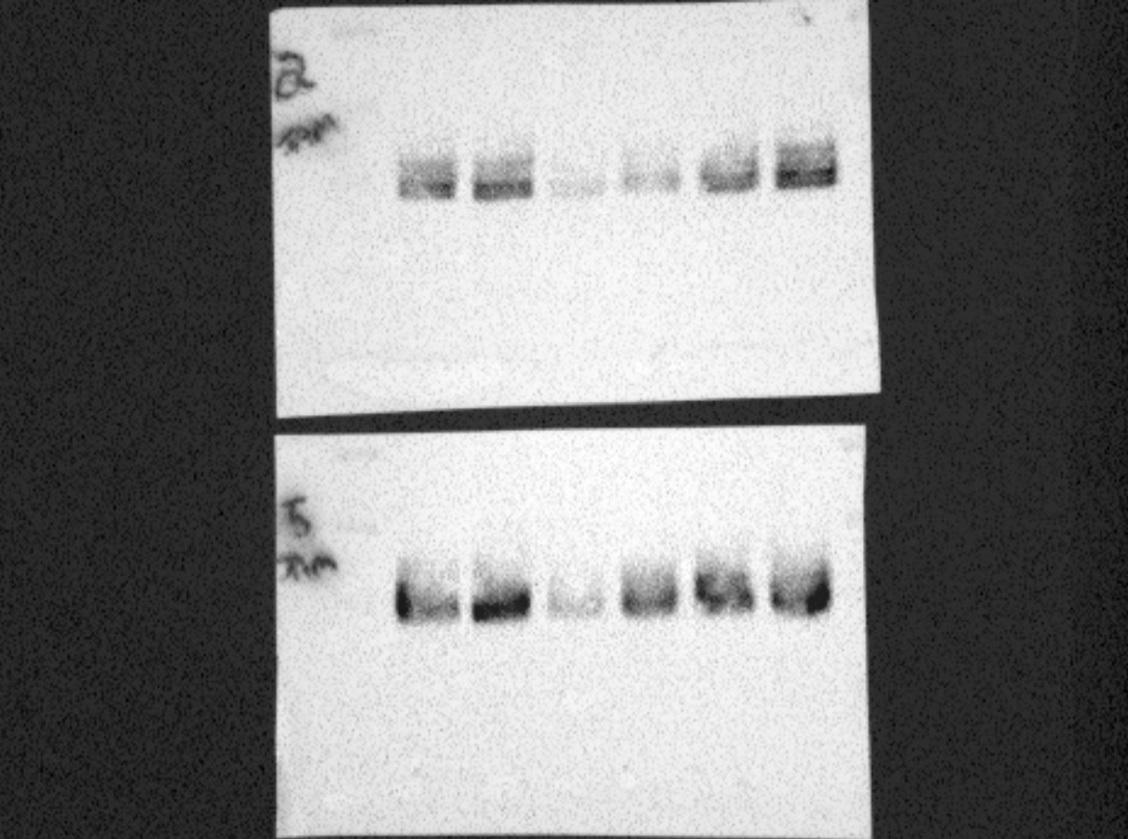  37-  50-  25-  kD:  25-  37- |

| **Fig 1d HER3** | **Original** |
| --- | --- |
| 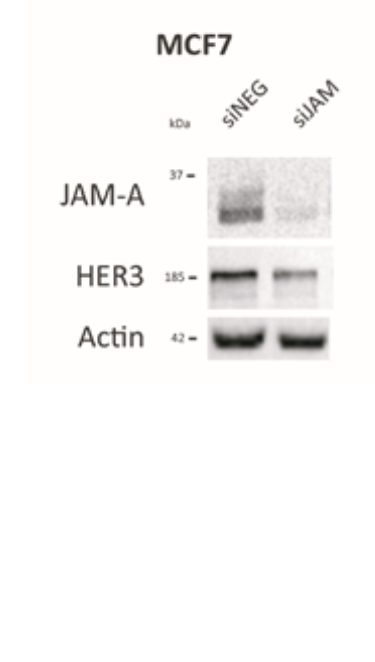 | 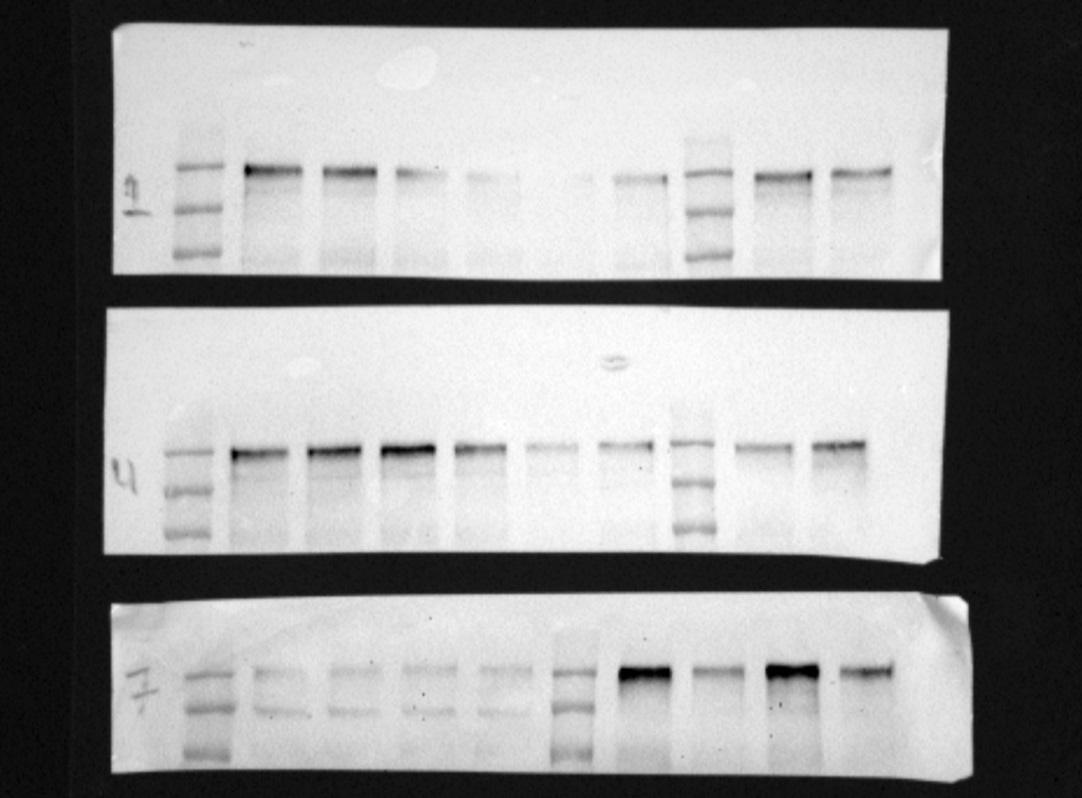  150-  100-  250-  kD: |

| **Fig 1d Actin** | **Original** |
| --- | --- |
| 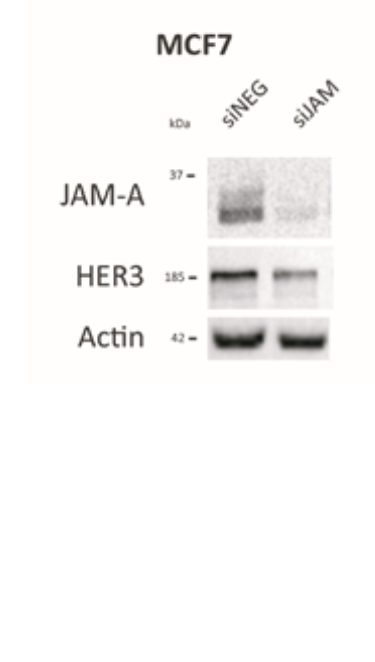 | 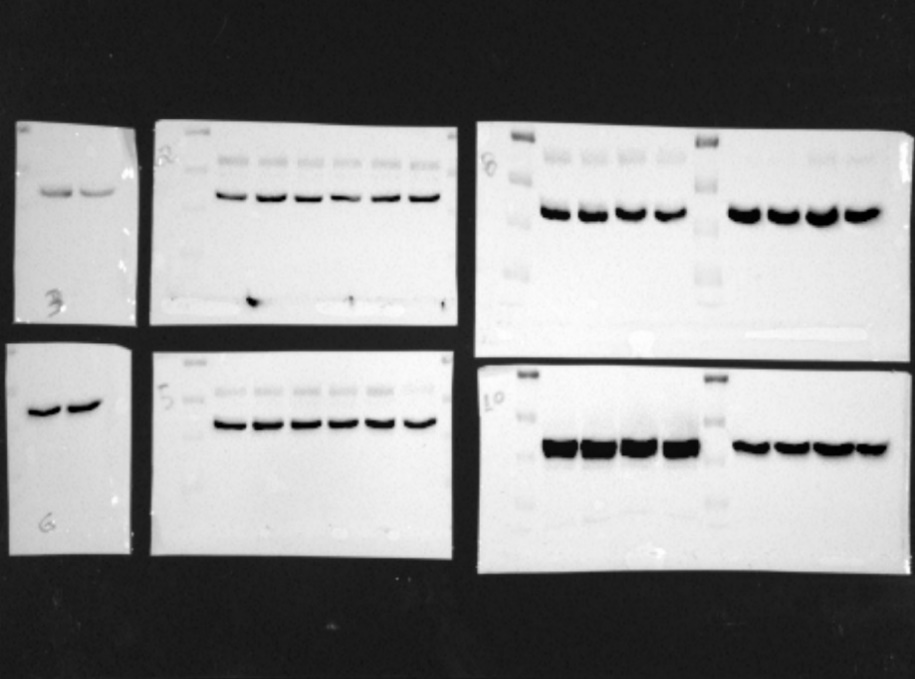  75-  37-  50- |

**Figure 2**

| **Fig 2a Actin** | **Original**  kD: |
| --- | --- |
| 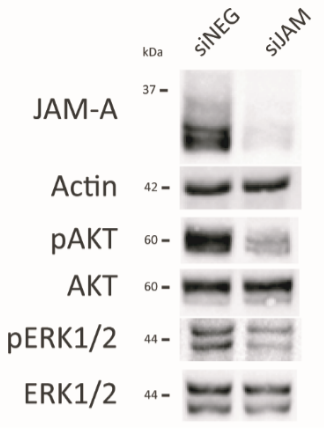 | 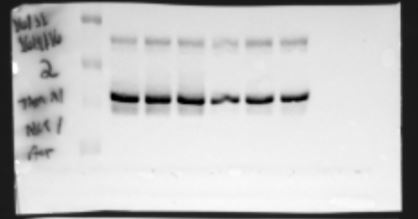  75-  50-  37-  25- |

| **Fig 2a JAM-A** | **Original** |
| --- | --- |
| 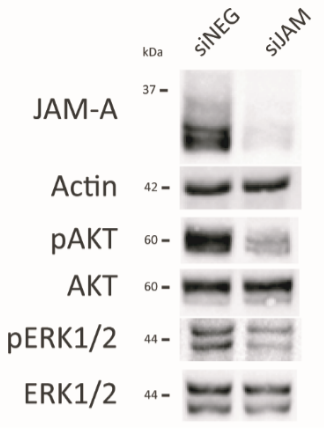 | 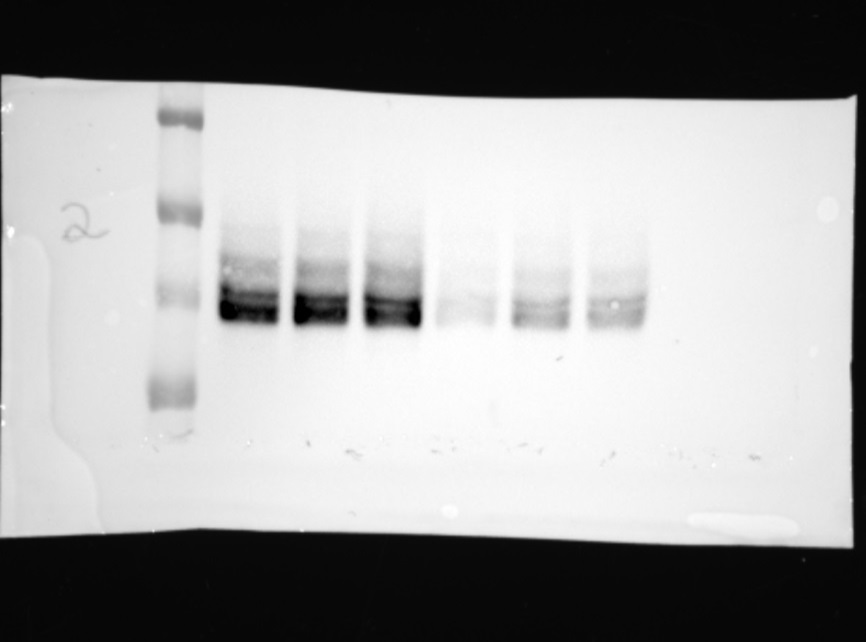  kD:  37-  25-  50-  75- |

| **Fig 2a pAKT** | **Original** |
| --- | --- |
| 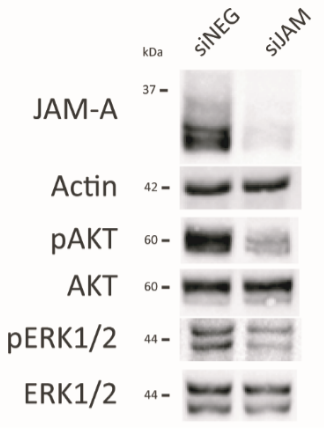 | 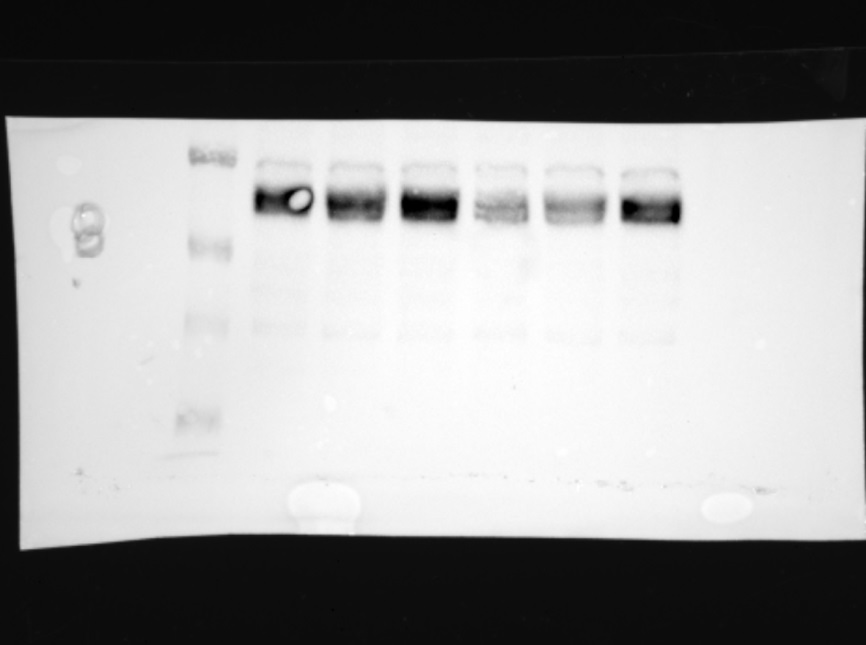  50-  25-  37-  kD:  75- |

| **Fig 2a AKT** | **Original** |
| --- | --- |
| 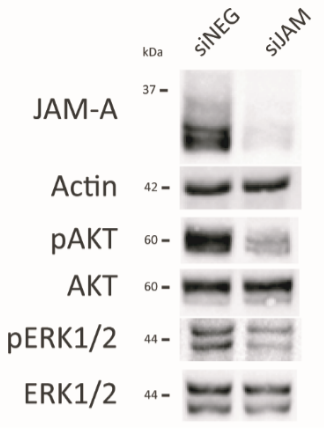 | 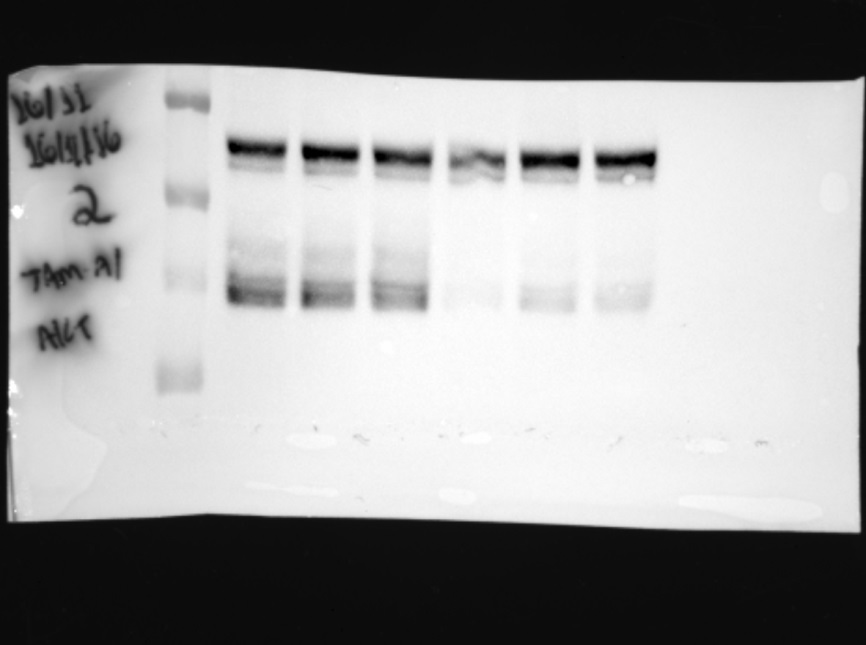  kD  75-  50-  37-  25- |

| **Fig 2a pERK 1/2** | **Original**  kD: |
| --- | --- |
| 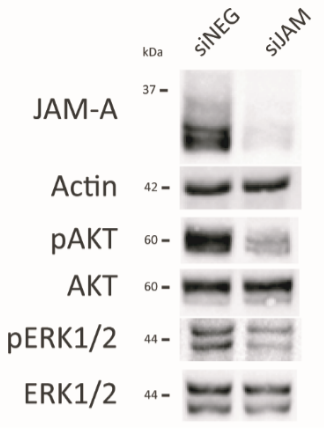 | 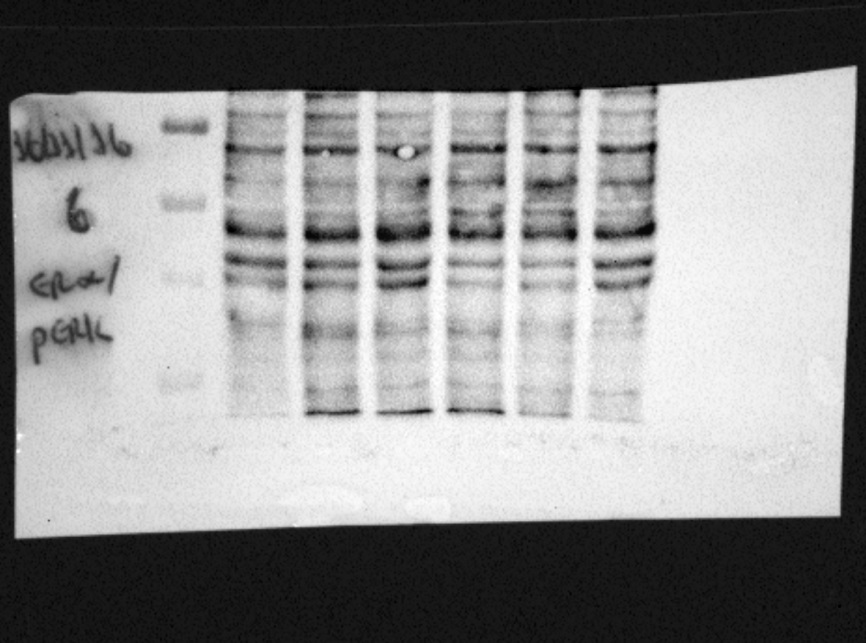  25-  37-  50-  75- |

| **Fig 2a ERK 1/2** | **Original**  75- |
| --- | --- |
| 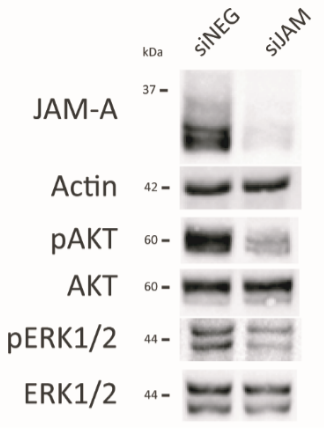 | 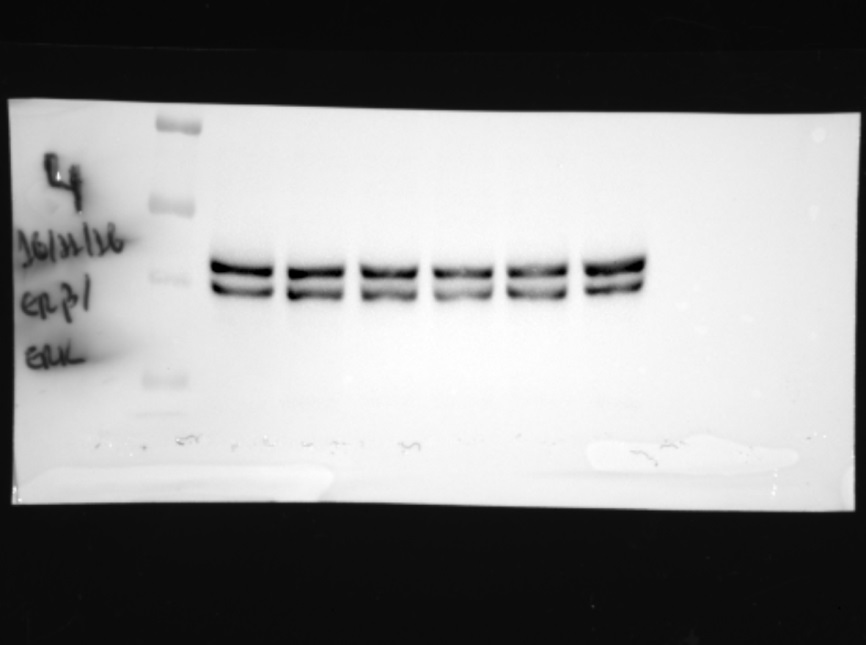  20-  25-  37-  50- |

| **Fig 2b Actin** | **Original** |
| --- | --- |
| 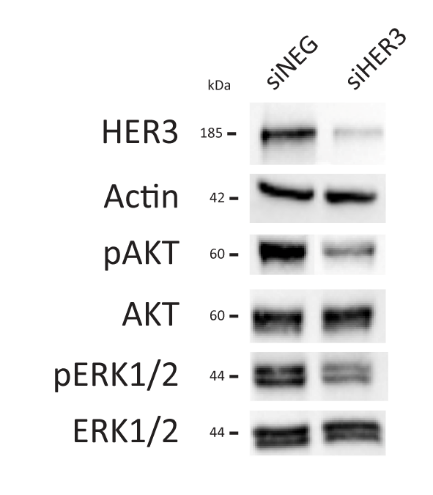 | 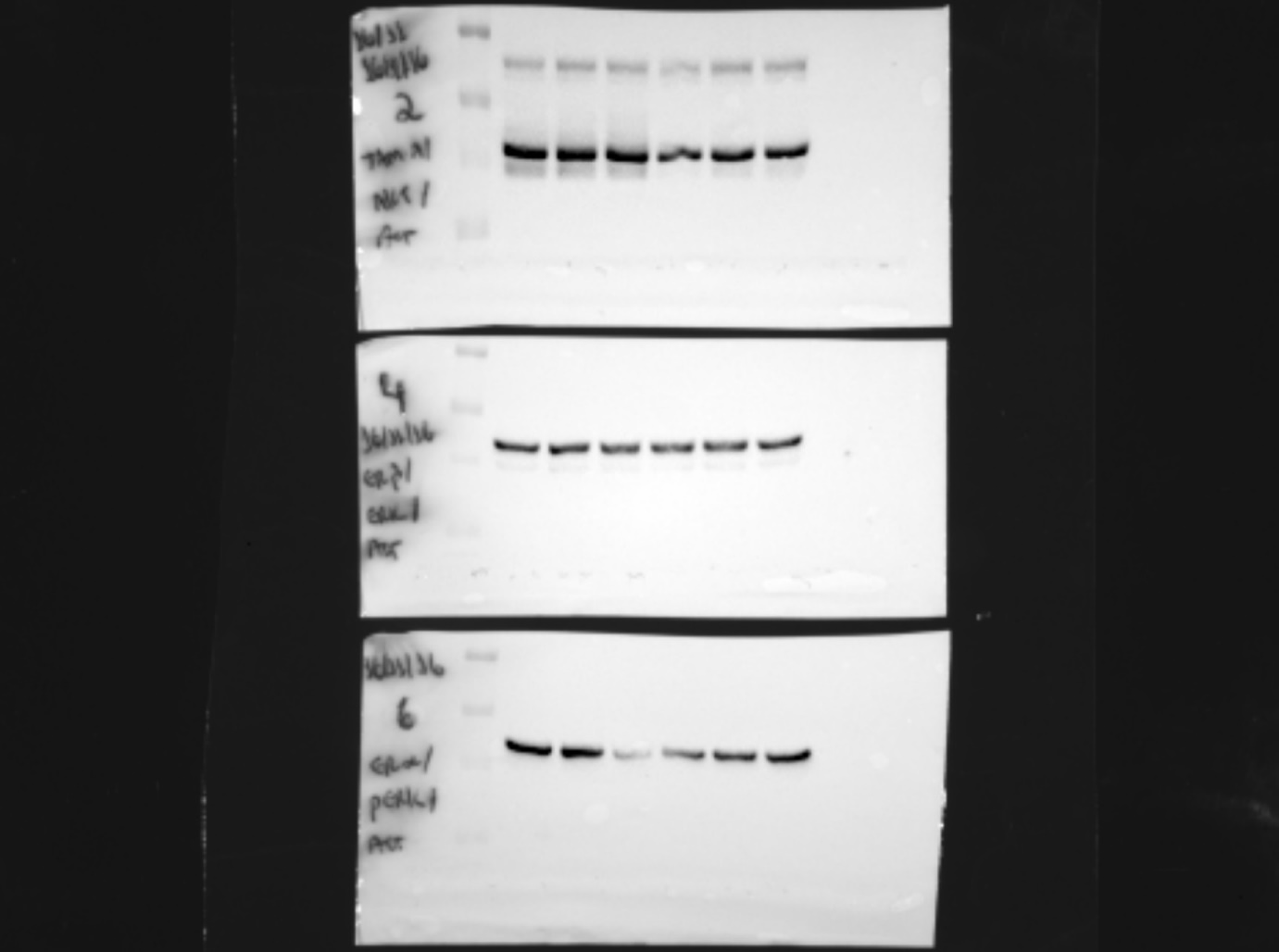  25-  37-  50-  75- |

| **Fig 2b HER3** | **Original** |
| --- | --- |
| 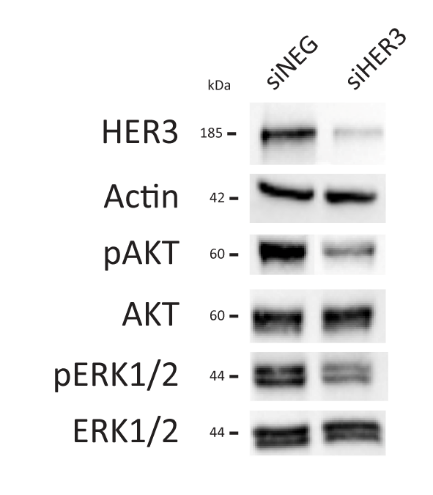 | 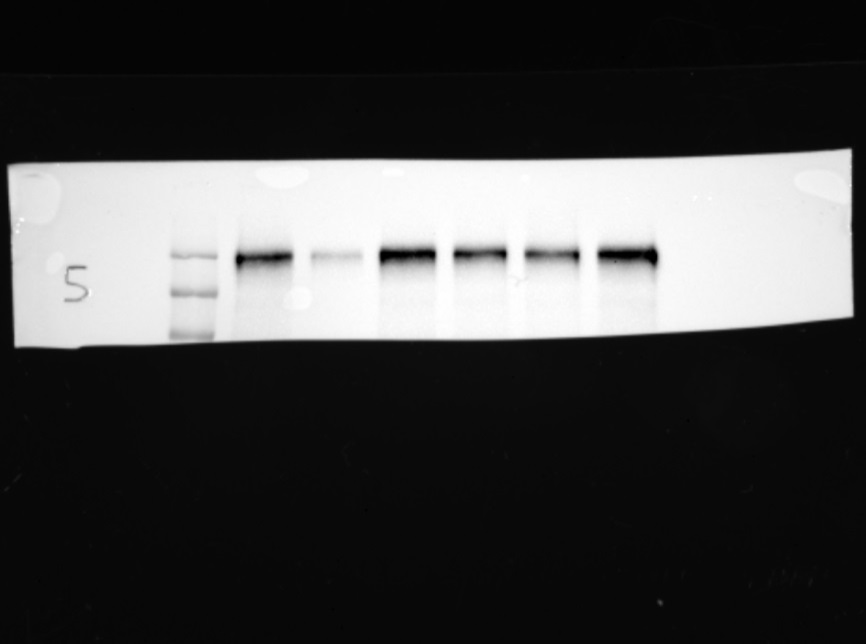  150-  250-  100-  kD: |

| **Fig 2b pAKT** | **Original** |
| --- | --- |
| 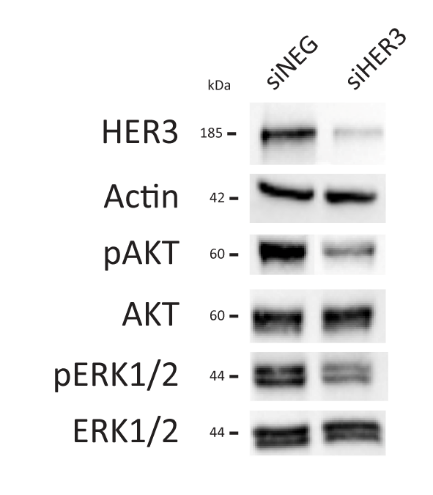 | 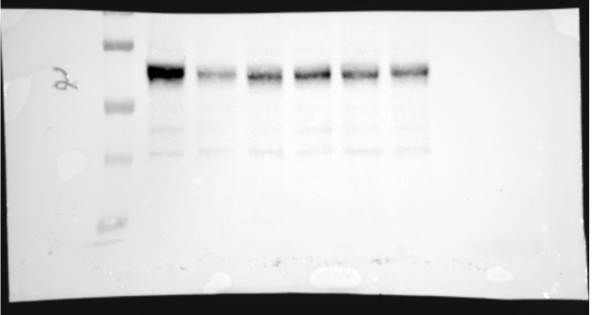  20-  25-  37-  50-  75-  kD: |

| **Fig 2b AKT** | **Original** |
| --- | --- |
| 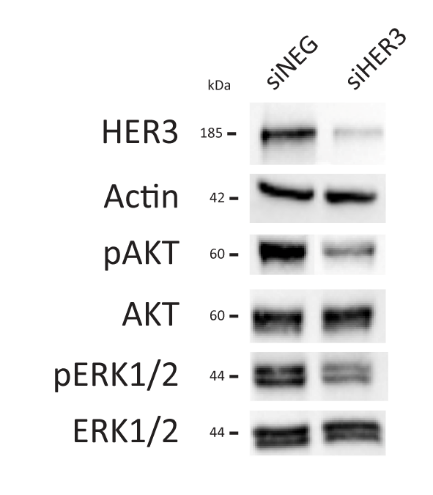 | 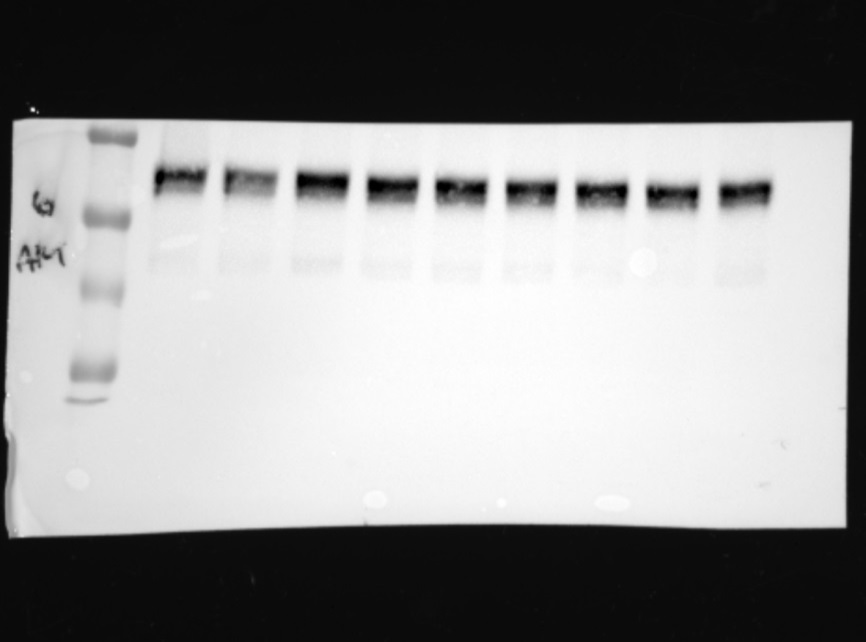  20-  25-  37-  50-  75-  kD: |

| **Fig 2b pERK 1/2** | **Original** |
| --- | --- |
| 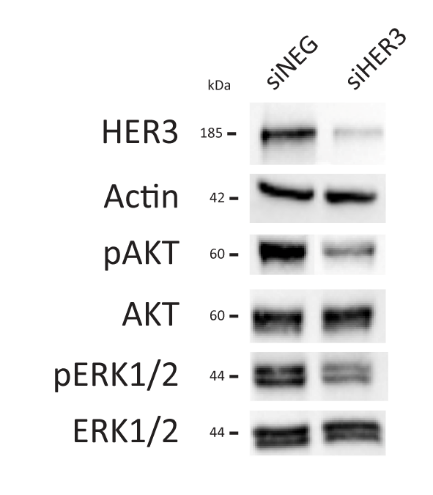 | 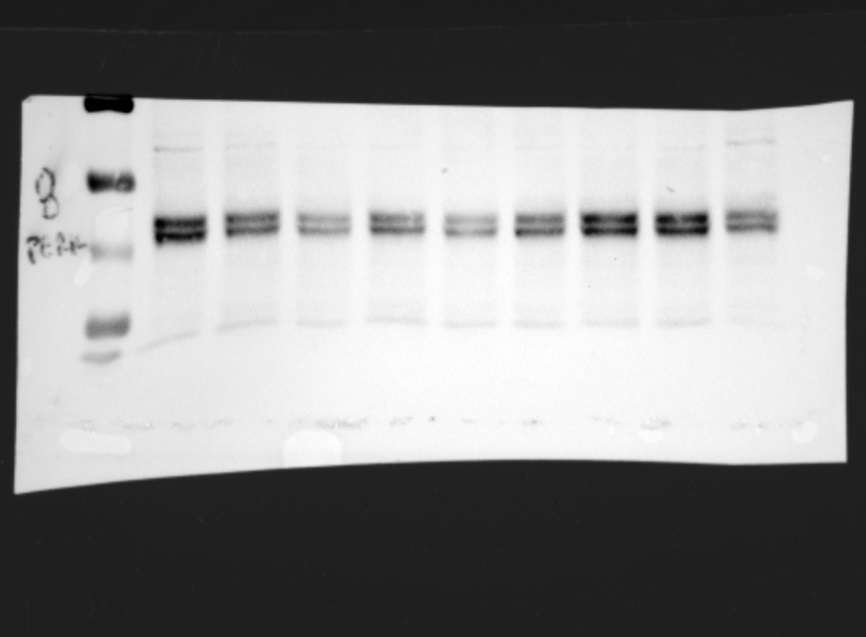  **75-**  kD:  37-  **50-**  25-  20- |

| **Fig 2b ERK 1/2** | **Original** |
| --- | --- |
| 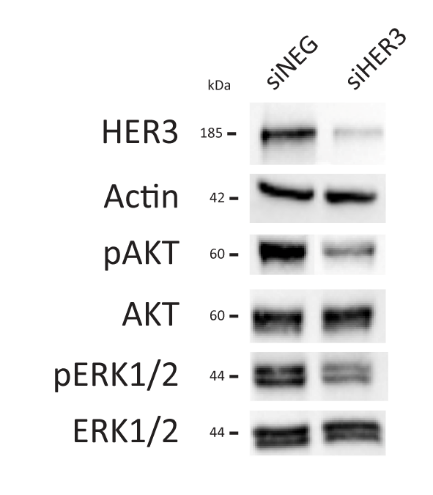 | 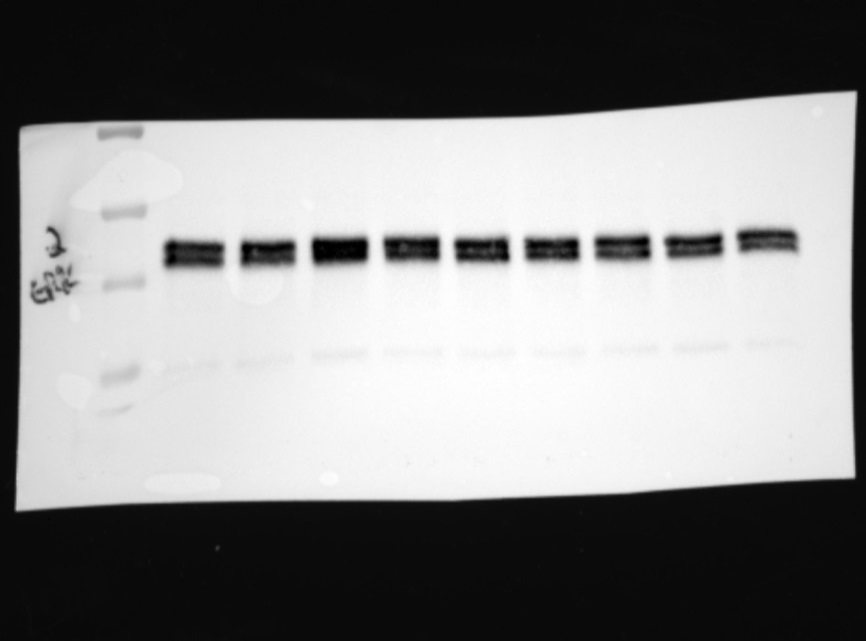  20-  25-  37-  50-  75-  kD: |

| **Fig 2e Actin** | **Original**  75- |
| --- | --- |
| 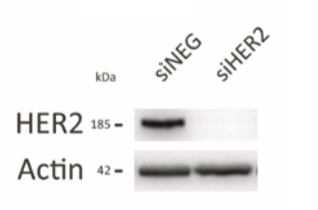 | 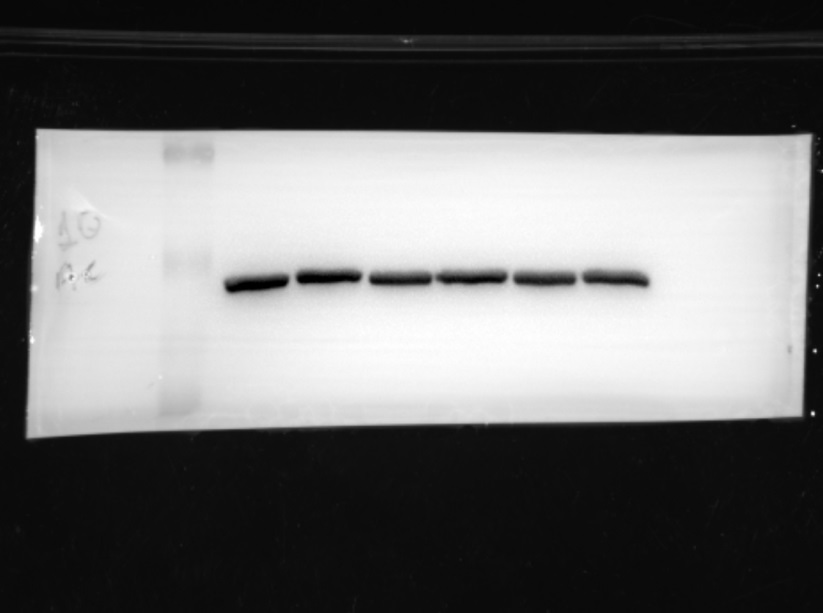  37-  20-  50- |

| **Fig 2e HER2** | **Original** |
| --- | --- |
| 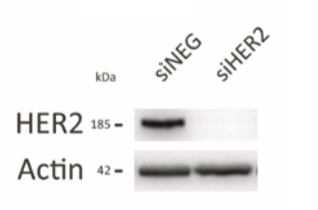 | 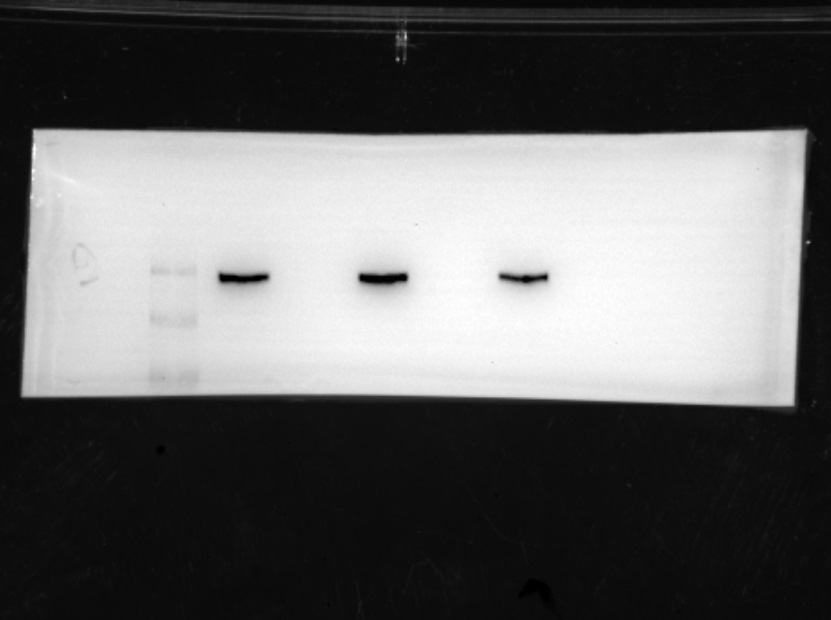  kD:  250-  185-  100- |

**Figure 3**

| **Fig 3b Actin** | **Original** |
| --- | --- |
| 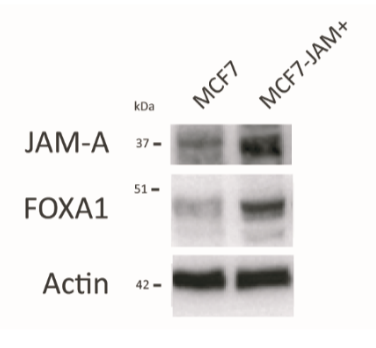 | kD:  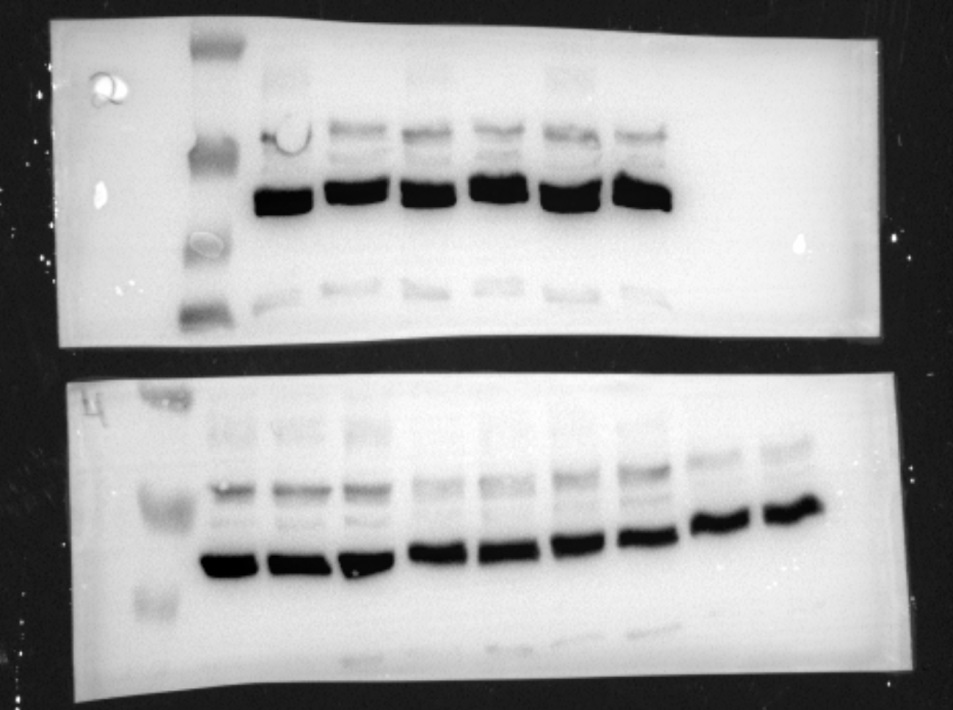  37-  50-  75- |

| **Fig 3b FOXA1** | **Original**  kD: |
| --- | --- |
| 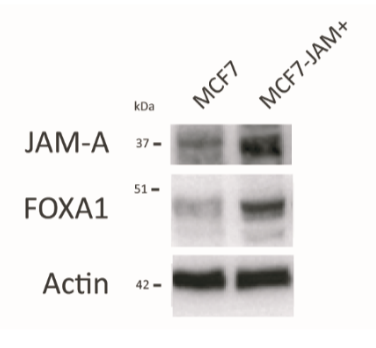 | -75  75-  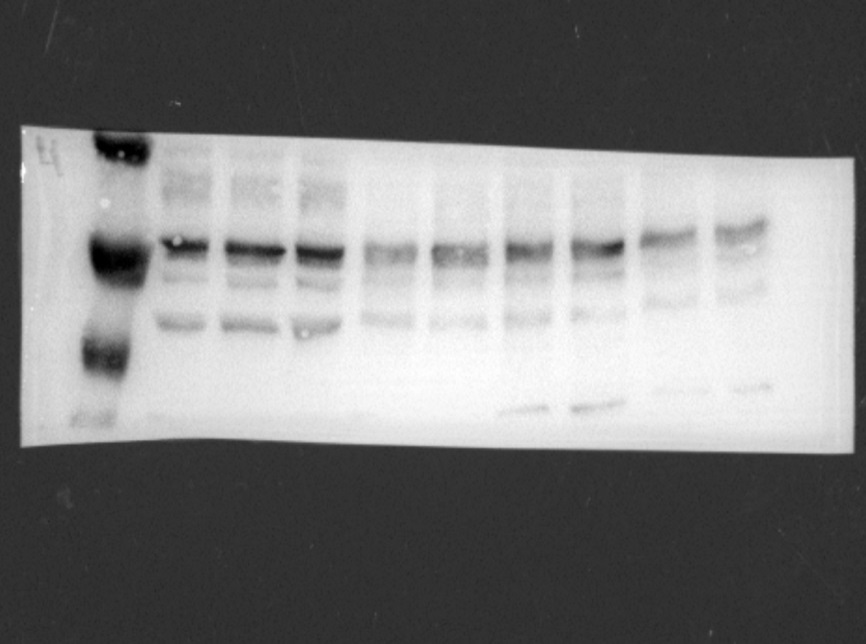  -50  -37  -25 |

kD:

-50

| **Fig 3b JAM-A** | **Original** |
| --- | --- |
| 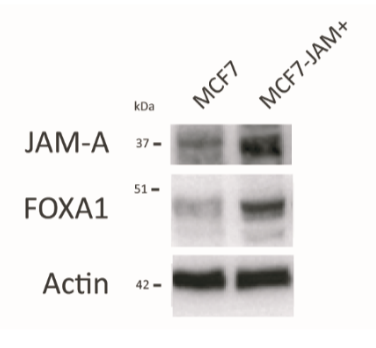 | 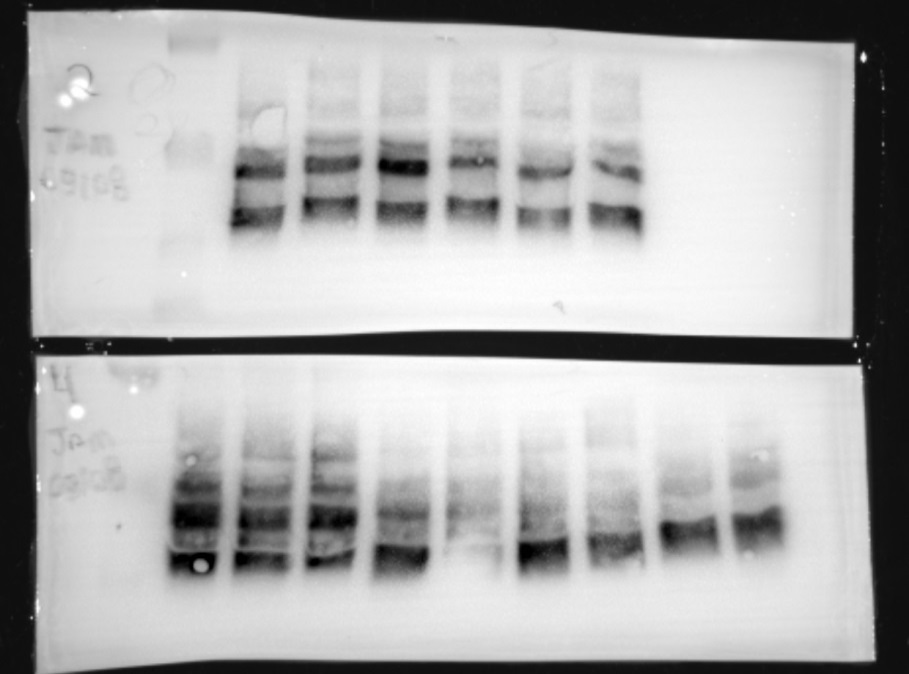  -25-  -37 |

| **Fig 3d JAM-A** | **Original** |
| --- | --- |
| 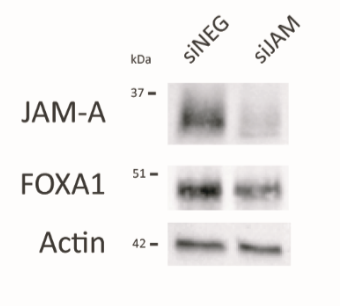 | 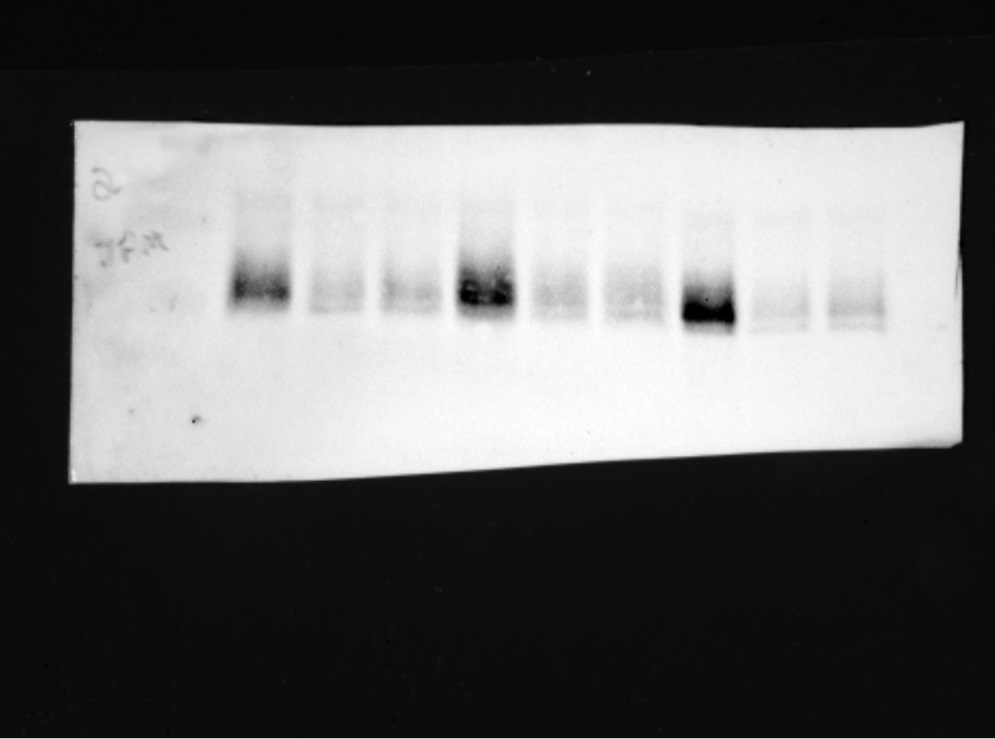  kD:  25-  37-  50- |

| **Fig 3d FOXA1** | **Original** |
| --- | --- |
| 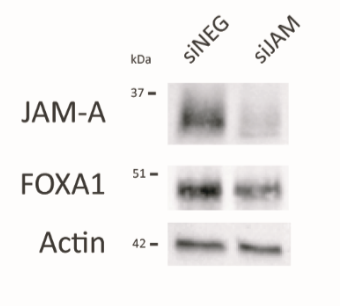 | 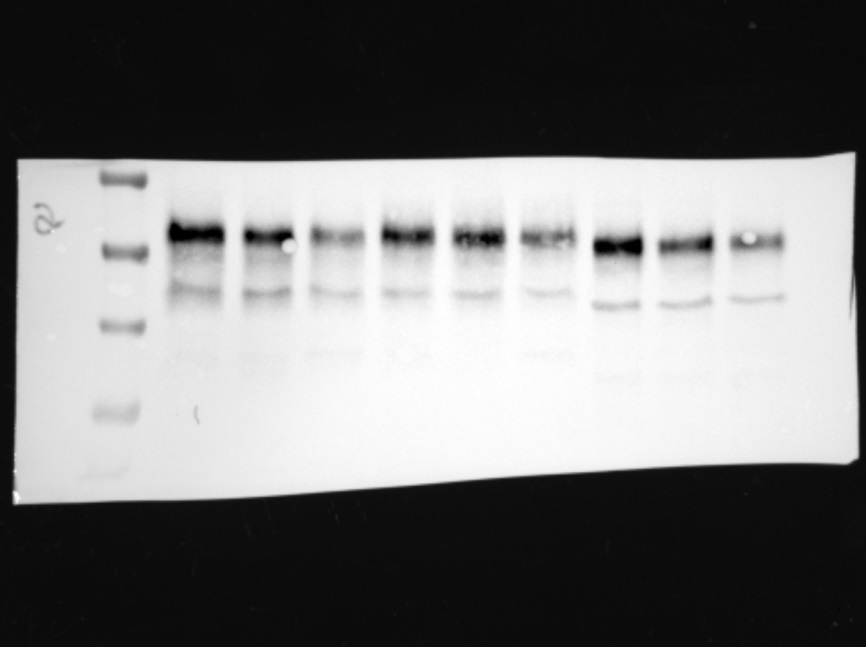  20-  25-  37-  50-  75-  kD: |

| **Fig 3d Actin** | **Original** |
| --- | --- |
| 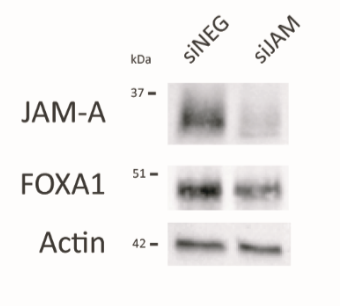 | 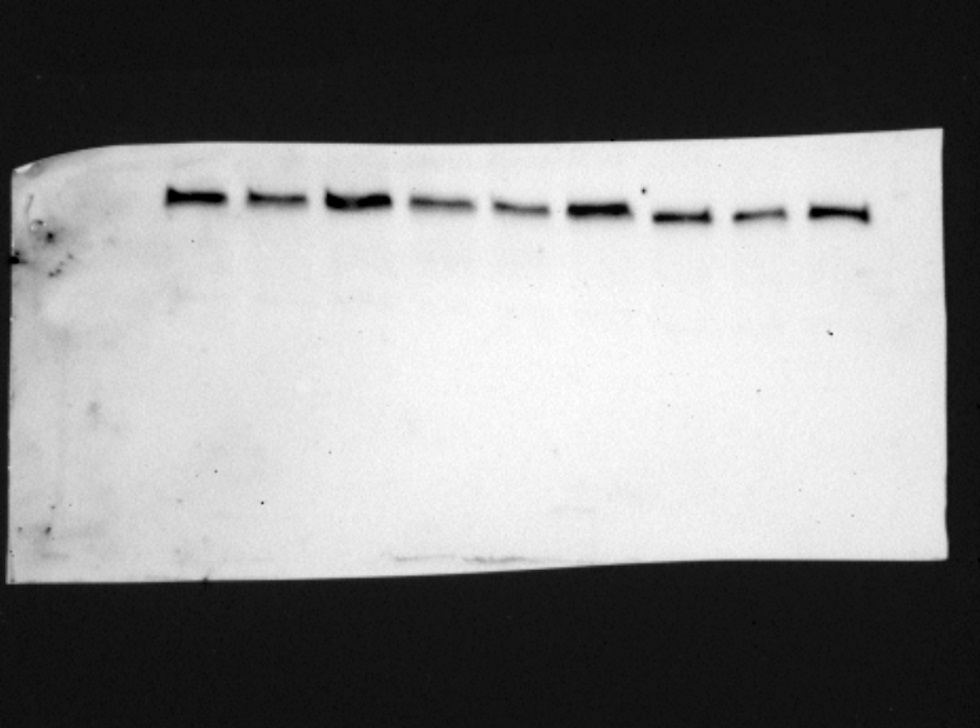  25-  37-  kD: |

**Figure 4**

| **Fig 4b Actin** | **Original** |
| --- | --- |
| 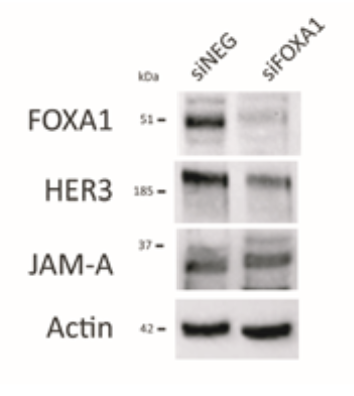 | 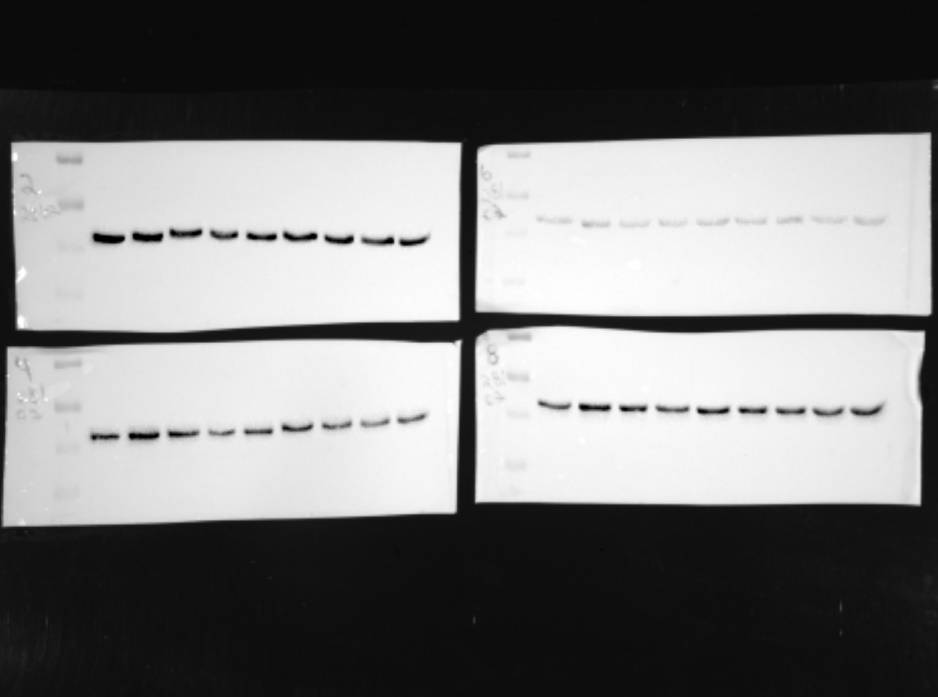  25-  37-  50-  75- |

| **Fig 4b FOXA1** | **Original**  -75 |
| --- | --- |
| 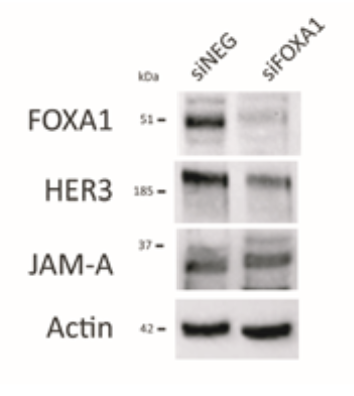 | 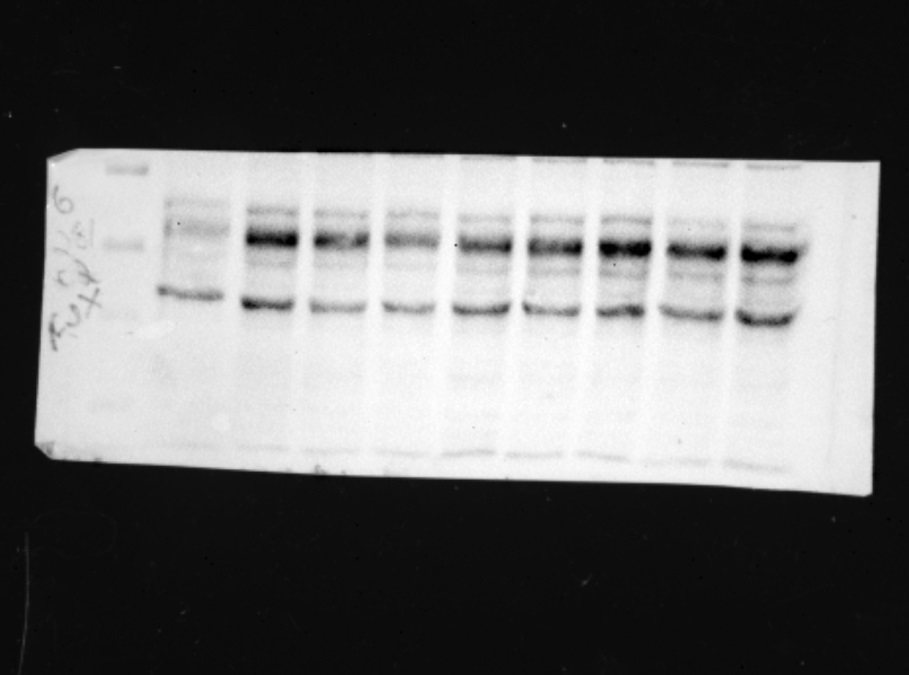  -37  -25 |

| **Fig 4b HER3** | **Original** |
| --- | --- |
| 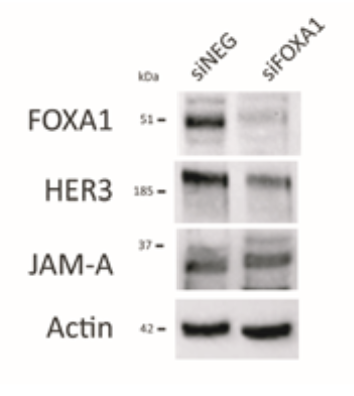 | 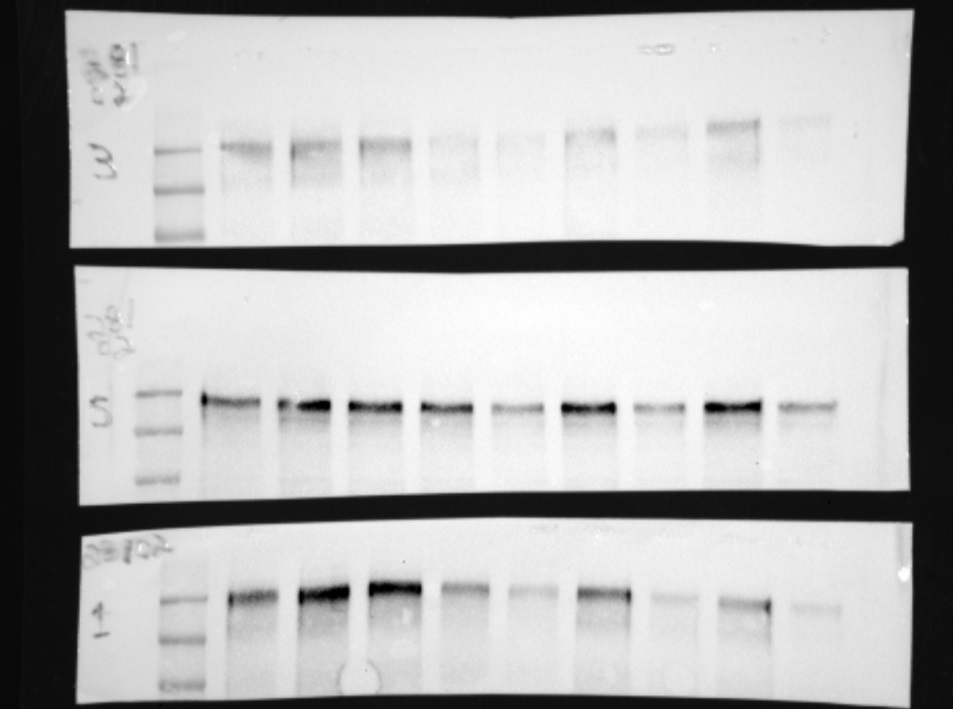  -100  -150  -250 |

| **Fig 4b JAM-A** | **Original** |
| --- | --- |
| 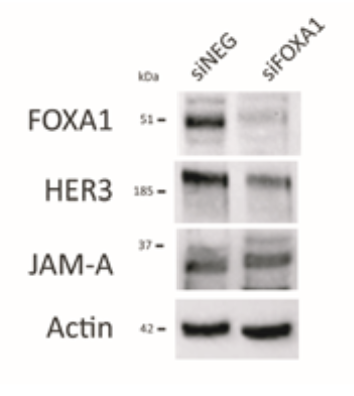 | 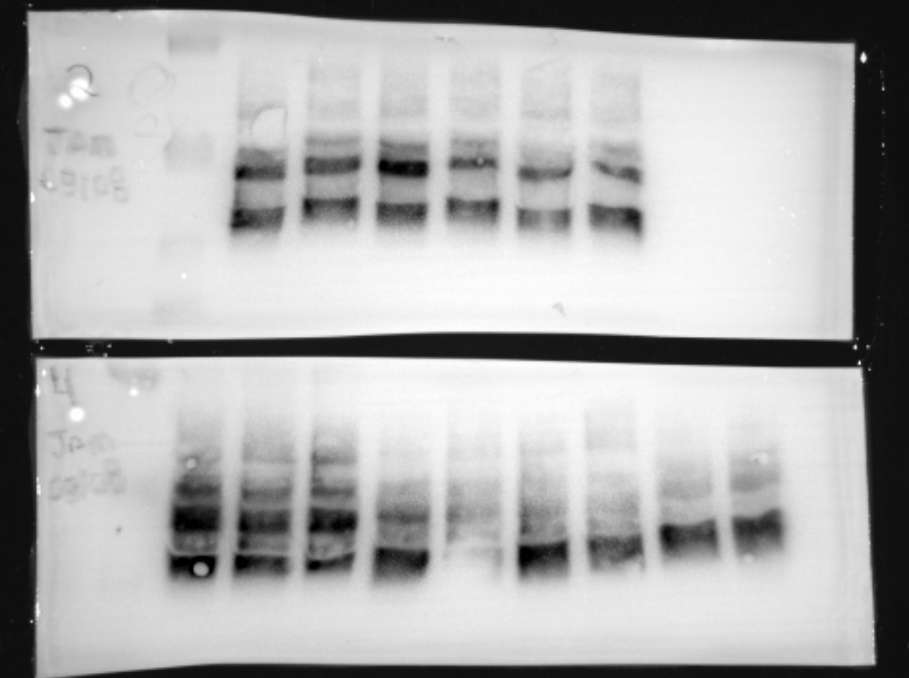  20-  25-  37-  50- |

**Figure 5**

| **Fig 5d Actin** | **Original** |
| --- | --- |
| 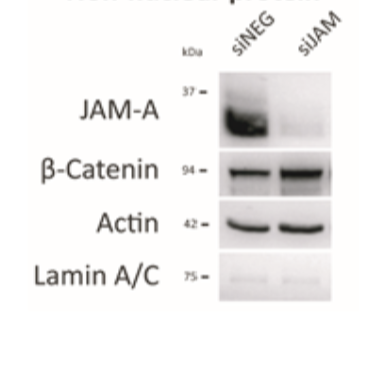 | kD:  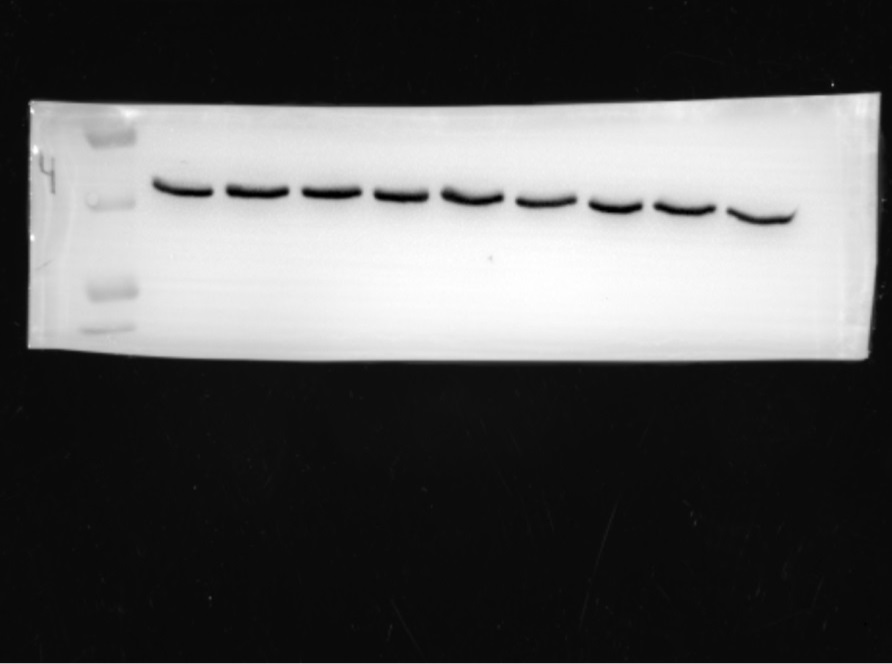  37-  50-  20-  25- |

| **Fig 5d B-Catenin** | **Original** |
| --- | --- |
| 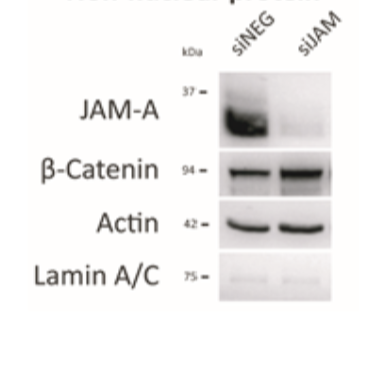 | 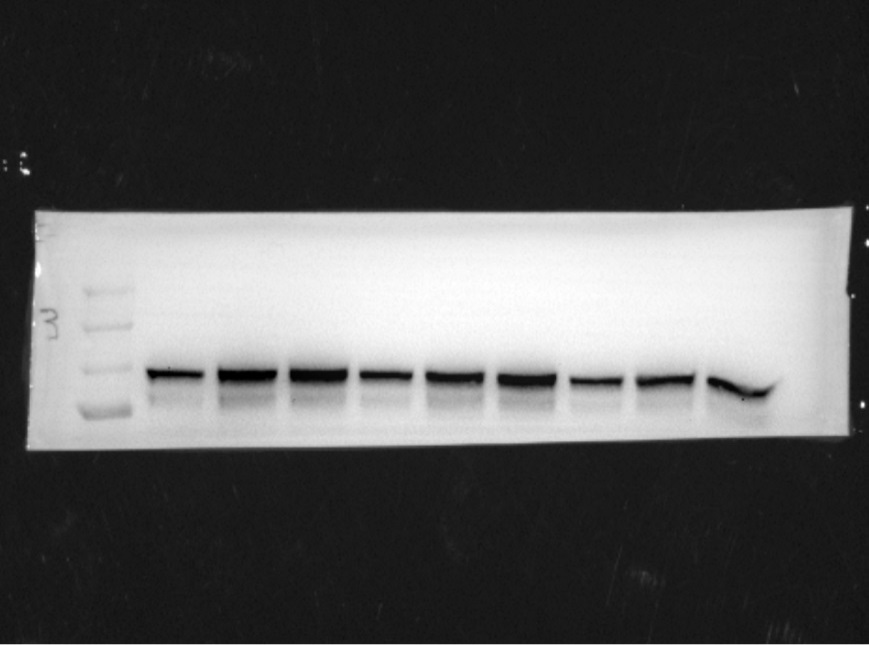  150-  75-  100-  250-  kD: |

| **Fig 5d JAM-A** | **Original**  kD: |
| --- | --- |
| 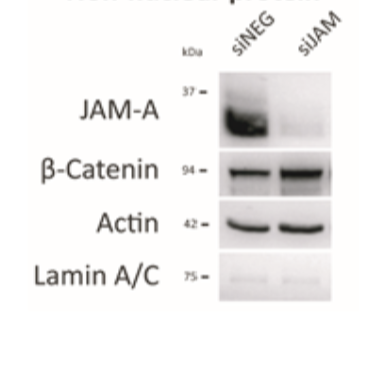 | 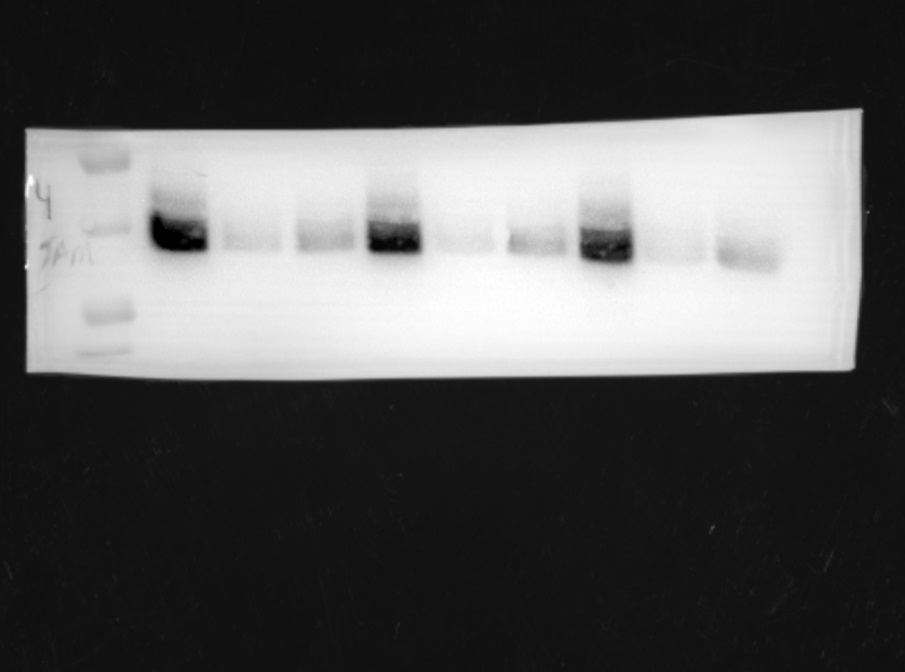  25-  20-  37-  50- |

| **Fig 5d Lamin A/C** | **Original** |
| --- | --- |
| 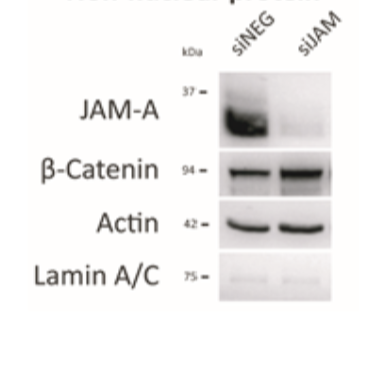 | kD:  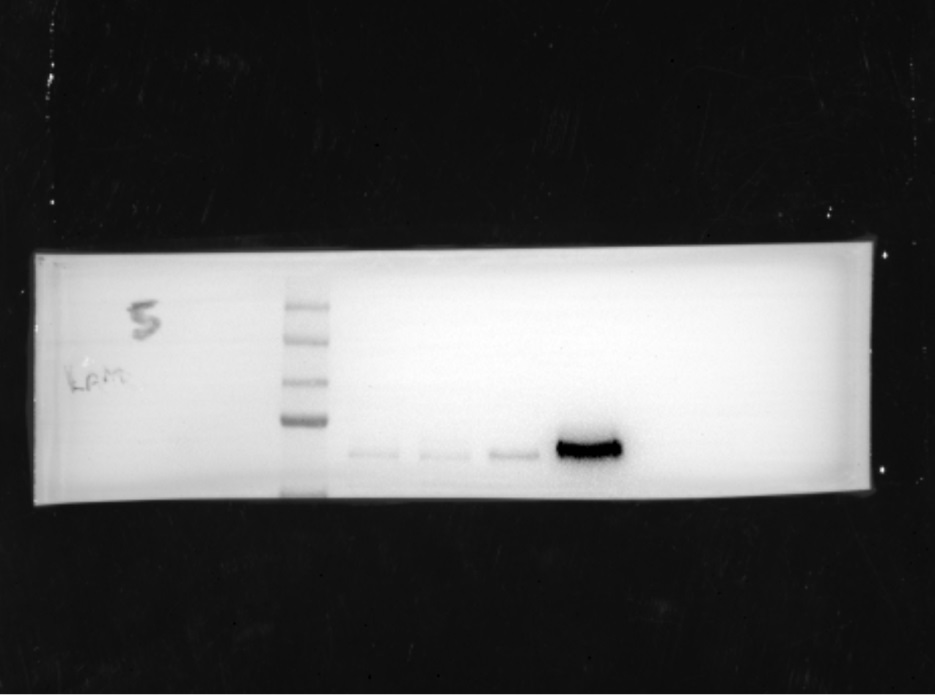  50-  150-  250-  75-  100- |

| **Fig 5e B-Catenin** | **Original** |
| --- | --- |
| 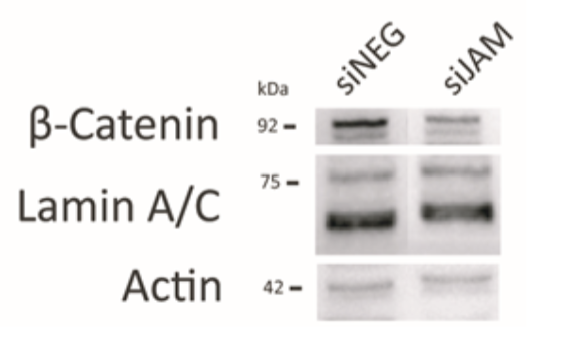 | 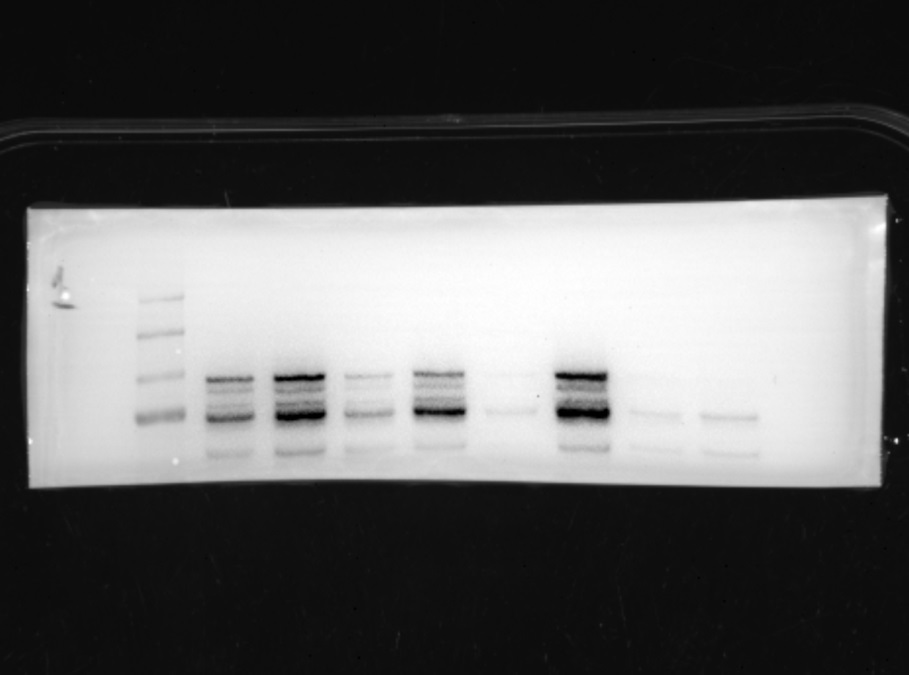  250-  75-  100-  150-  kD: |

| **Fig 5e Lamin A/C** | **Original** |
| --- | --- |
| 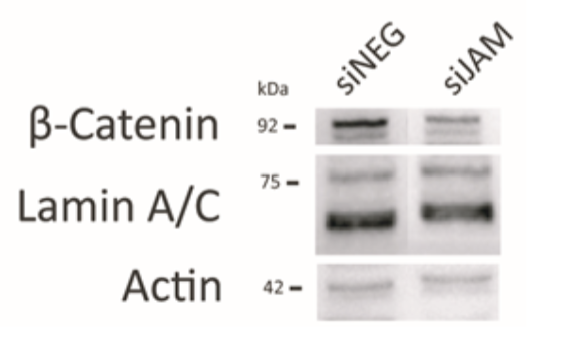  250-  75-  100-  150- | 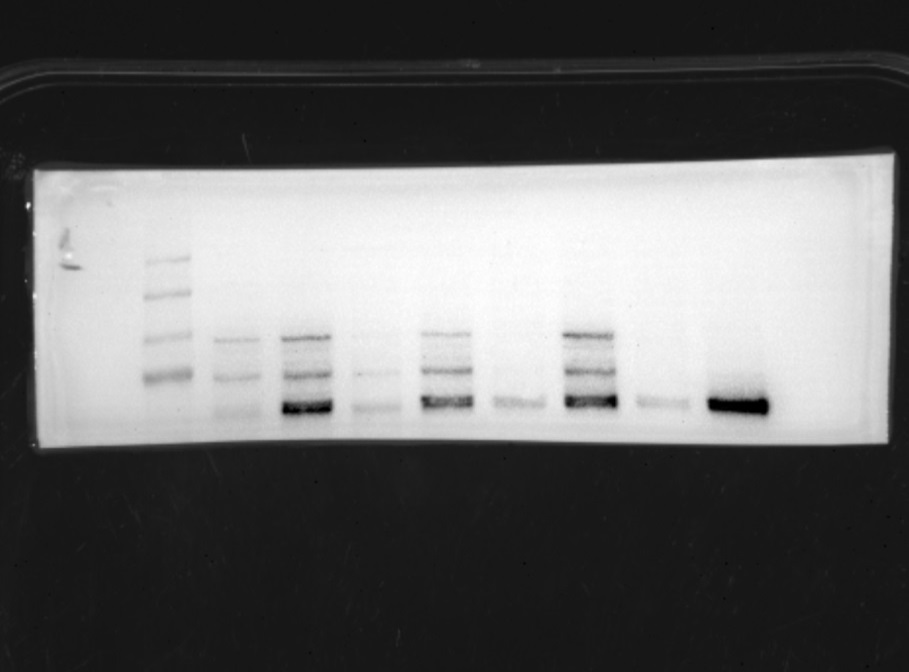  kD: |

| **Fig 5e Actin** | **Original** |
| --- | --- |
| 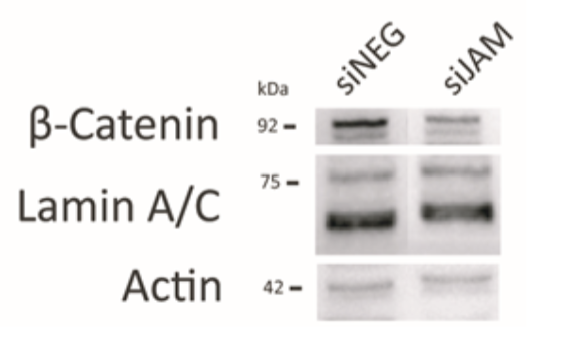  75-  100- | 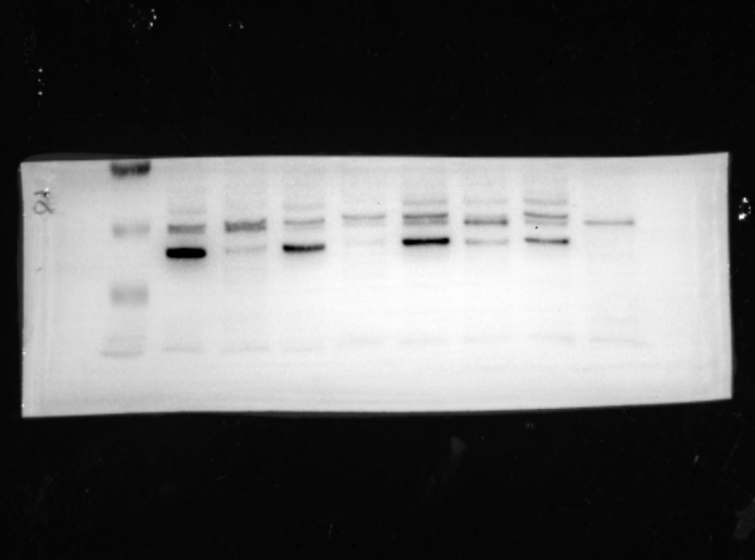  25-  37-  50-  20-  kD: |

| **Fig 5f B-Catenin** | **Original** |
| --- | --- |
| 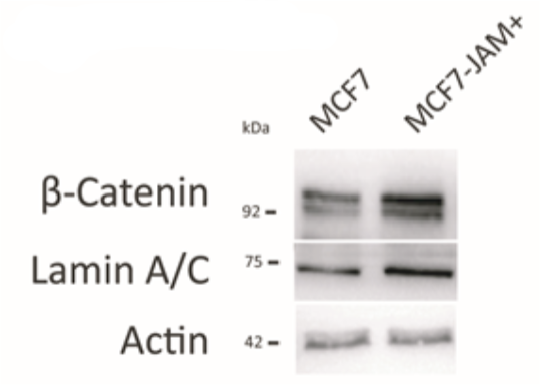  250-  100-  75- | kD:  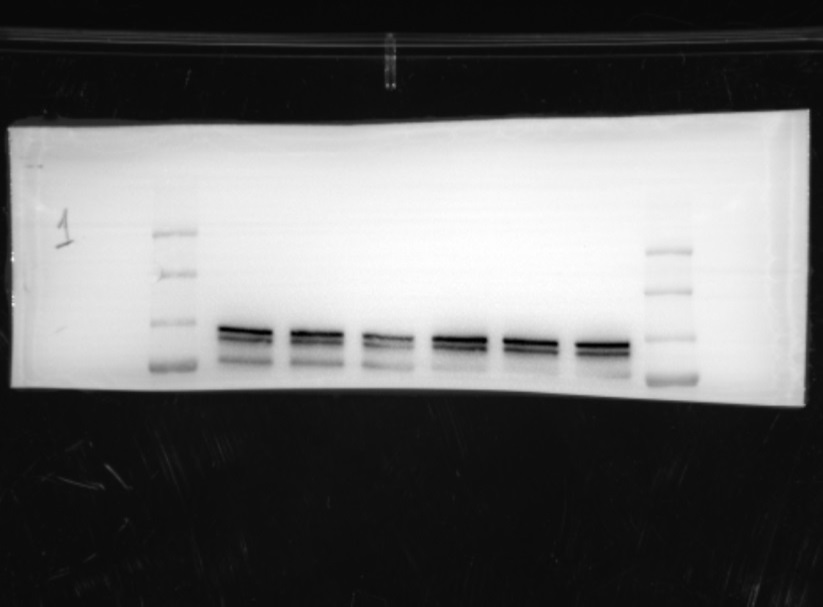  150- |

| **Fig 5f Lamin A/C** | **Original** |
| --- | --- |
| 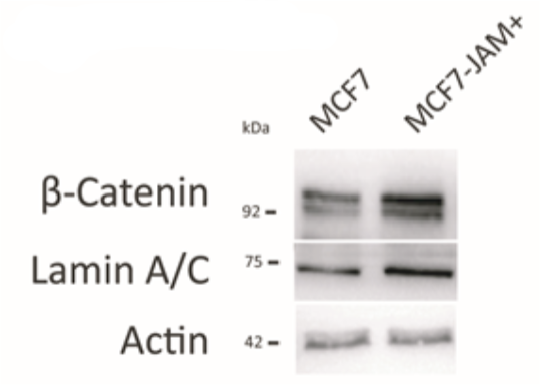  20-  25-  37- | 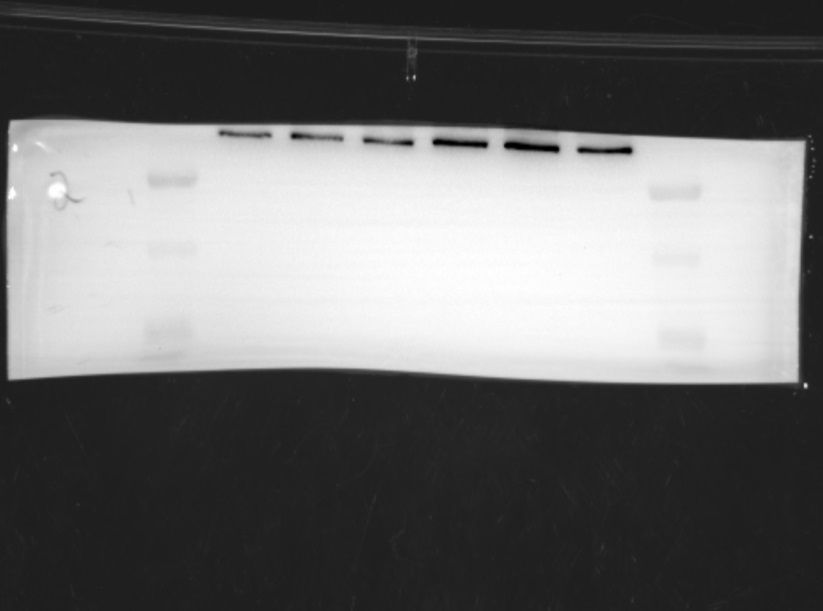  50-  kD: |

| **Fig 5f Actin** | **Original** |
| --- | --- |
| 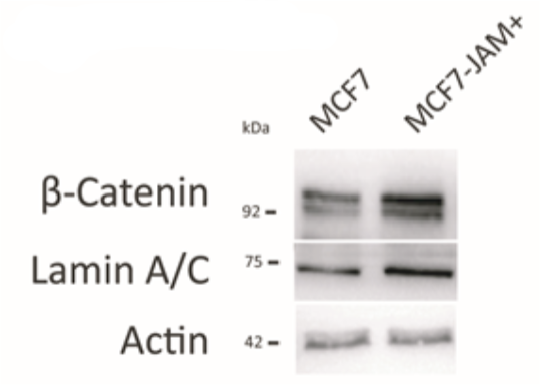 | 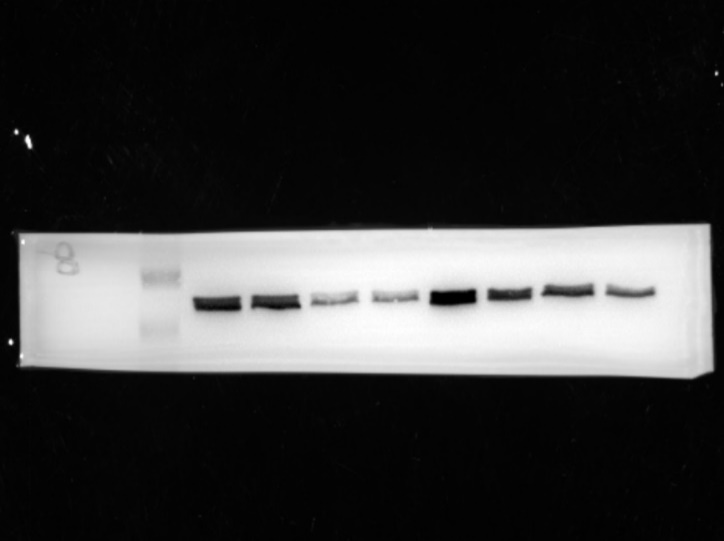  kD:  37-  50- |
