# Supplementary material for: Human Epidermal Growth Factor Receptor-3 Expression Is Regulated at Transcriptional Level in Breast Cancer Settings by Junctional Adhesion Molecule-A via a Pathway Involving Beta-Catenin and FOXA1
Source: Cancers (Basel). 2021 Feb 19;13(4):871. doi: 10.3390/cancers13040871 (PMC7922773; doi:10.3390/cancers13040871)
Supplement: Supplementary file 1 [file cancers-13-00871-s001.zip › Supplementary Tables.docx]

**Supplementary Table S1. Genes coexpressed with HER3 in breast cancer.**

Weighted gene co-expression network analysis (WGCNA; Clarke C et al., *Carcinogenesis*. 2013;34(10):2300-8.) was used to identify genes co-expressed with HER3 across 2,342 breast cancer samples from 13 independent gene expression studies. A correlation coefficient of > or <0.5 was chosen as the cut-off point, and 37 genes exhibited statistically significant correlations. Of those 37 genes, the disease-free survival hazard ratios (DFS-HR) of 8 were also statistically significant (shaded rows; p<0.05).

| **AffyID** | **EntrezID** | **Gene Symbol** | **Gene Title** | **Correlation** | **DFS HR** | **P-value** |
| --- | --- | --- | --- | --- | --- | --- |
| [202625_at](http://glados.ucd.ie/Coexpression/display_network.php?gene_name=4067&survival_type=dfs) | [4067](http://www.ncbi.nlm.nih.gov/gene?term=4067) | [LYN](http://coxpresdb.jp/data/locus/4067.shtml) | v-yes-1 Yamaguchi sarcoma viral related oncogene homolog | -0.5 | 1.04 | 6.53E-01 |
| [204508_s_at](http://glados.ucd.ie/Coexpression/display_network.php?gene_name=771&survival_type=dfs) | [771](http://www.ncbi.nlm.nih.gov/gene?term=771) | [CA12](http://coxpresdb.jp/data/locus/771.shtml) | carbonic anhydrase XII | 0.52 | 0.87 | 8.93E-02 |
| [209603_at](http://glados.ucd.ie/Coexpression/display_network.php?gene_name=2625&survival_type=dfs) | [2625](http://www.ncbi.nlm.nih.gov/gene?term=2625) | [GATA3](http://coxpresdb.jp/data/locus/2625.shtml) | GATA binding protein 3 | 0.56 | 0.88 | 1.06E-01 |
| [205225_at](http://glados.ucd.ie/Coexpression/display_network.php?gene_name=2099&survival_type=dfs) | [2099](http://www.ncbi.nlm.nih.gov/gene?term=2099) | [ESR1](http://coxpresdb.jp/data/locus/2099.shtml) | estrogen receptor 1 | 0.51 | 0.88 | 1.29E-01 |
| [204667_at](http://glados.ucd.ie/Coexpression/display_network.php?gene_name=3169&survival_type=dfs) | [3169](http://www.ncbi.nlm.nih.gov/gene?term=3169) | [FOXA1](http://coxpresdb.jp/data/locus/3169.shtml) | forkhead box A1 | 0.59 | 0.83 | 2.61E-02 |
| [209602_s_at](http://glados.ucd.ie/Coexpression/display_network.php?gene_name=2625&survival_type=dfs) | [2625](http://www.ncbi.nlm.nih.gov/gene?term=2625) | [GATA3](http://coxpresdb.jp/data/locus/2625.shtml) | GATA binding protein 3 | 0.55 | 0.88 | 1.30E-01 |
| [218211_s_at](http://glados.ucd.ie/Coexpression/display_network.php?gene_name=79083&survival_type=dfs) | [79083](http://www.ncbi.nlm.nih.gov/gene?term=79083) | [MLPH](http://coxpresdb.jp/data/locus/79083.shtml) | melanophilin | 0.58 | 0.82 | 1.71E-02 |
| [210735_s_at](http://glados.ucd.ie/Coexpression/display_network.php?gene_name=771&survival_type=dfs) | [771](http://www.ncbi.nlm.nih.gov/gene?term=771) | [CA12](http://coxpresdb.jp/data/locus/771.shtml) | carbonic anhydrase XII | 0.5 | 0.84 | 3.98E-02 |
| [209604_s_at](http://glados.ucd.ie/Coexpression/display_network.php?gene_name=2625&survival_type=dfs) | [2625](http://www.ncbi.nlm.nih.gov/gene?term=2625) | [GATA3](http://coxpresdb.jp/data/locus/2625.shtml) | GATA binding protein 3 | 0.55 | 0.91 | 2.28E-01 |
| [200670_at](http://glados.ucd.ie/Coexpression/display_network.php?gene_name=7494&survival_type=dfs) | [7494](http://www.ncbi.nlm.nih.gov/gene?term=7494) | [XBP1](http://coxpresdb.jp/data/locus/7494.shtml) | X-box binding protein 1 | 0.52 | 0.8 | 7.47E-03 |
| [220192_x_at](http://glados.ucd.ie/Coexpression/display_network.php?gene_name=25803&survival_type=dfs) | [25803](http://www.ncbi.nlm.nih.gov/gene?term=25803) | [SPDEF](http://coxpresdb.jp/data/locus/25803.shtml) | SAM pointed domain containing ets transcription factor | 0.58 | 1.1 | 2.52E-01 |
| [208682_s_at](http://glados.ucd.ie/Coexpression/display_network.php?gene_name=10916&survival_type=dfs) | [10916](http://www.ncbi.nlm.nih.gov/gene?term=10916) | [MAGED2](http://coxpresdb.jp/data/locus/10916.shtml) | melanoma antigen family D, 2 | 0.55 | 0.98 | 7.97E-01 |
| [214404_x_at](http://glados.ucd.ie/Coexpression/display_network.php?gene_name=25803&survival_type=dfs) | [25803](http://www.ncbi.nlm.nih.gov/gene?term=25803) | [SPDEF](http://coxpresdb.jp/data/locus/25803.shtml) | SAM pointed domain containing ets transcription factor | 0.52 | 1.11 | 1.92E-01 |
| [213441_x_at](http://glados.ucd.ie/Coexpression/display_network.php?gene_name=25803&survival_type=dfs) | [25803](http://www.ncbi.nlm.nih.gov/gene?term=25803) | [SPDEF](http://coxpresdb.jp/data/locus/25803.shtml) | SAM pointed domain containing ets transcription factor | 0.53 | 1.13 | 1.38E-01 |
| [205074_at](http://glados.ucd.ie/Coexpression/display_network.php?gene_name=6584&survival_type=dfs) | [6584](http://www.ncbi.nlm.nih.gov/gene?term=6584) | [SLC22A5](http://coxpresdb.jp/data/locus/6584.shtml) | solute carrier family 22 (organic cation/carnitine transporter), member 5 | 0.5 | 0.89 | 1.56E-01 |
| [212099_at](http://glados.ucd.ie/Coexpression/display_network.php?gene_name=388&survival_type=dfs) | [388](http://www.ncbi.nlm.nih.gov/gene?term=388) | [RHOB](http://coxpresdb.jp/data/locus/388.shtml) | ras homolog family member B | 0.52 | 0.87 | 9.15E-02 |
| [212692_s_at](http://glados.ucd.ie/Coexpression/display_network.php?gene_name=987&survival_type=dfs) | [987](http://www.ncbi.nlm.nih.gov/gene?term=987) | [LRBA](http://coxpresdb.jp/data/locus/987.shtml) | LPS-responsive vesicle trafficking, beach and anchor containing | 0.52 | 0.88 | 1.07E-01 |
| [205597_at](http://glados.ucd.ie/Coexpression/display_network.php?gene_name=80736&survival_type=dfs) | [80736](http://www.ncbi.nlm.nih.gov/gene?term=80736) | [SLC44A4](http://coxpresdb.jp/data/locus/80736.shtml) | solute carrier family 44, member 4 | 0.54 | 0.82 | 1.96E-02 |
| [213627_at](http://glados.ucd.ie/Coexpression/display_network.php?gene_name=10916&survival_type=dfs) | [10916](http://www.ncbi.nlm.nih.gov/gene?term=10916) | [MAGED2](http://coxpresdb.jp/data/locus/10916.shtml) | melanoma antigen family D, 2 | 0.5 | 1.01 | 9.16E-01 |
| [204798_at](http://glados.ucd.ie/Coexpression/display_network.php?gene_name=4602&survival_type=dfs) | [4602](http://www.ncbi.nlm.nih.gov/gene?term=4602) | [MYB](http://coxpresdb.jp/data/locus/4602.shtml) | v-myb myeloblastosis viral oncogene homolog (avian) | 0.54 | 0.78 | 2.43E-03 |
| [209623_at](http://glados.ucd.ie/Coexpression/display_network.php?gene_name=64087&survival_type=dfs) | [64087](http://www.ncbi.nlm.nih.gov/gene?term=64087) | [MCCC2](http://coxpresdb.jp/data/locus/64087.shtml) | methylcrotonoyl-CoA carboxylase 2 (beta) | 0.51 | 0.88 | 1.32E-01 |
| [218966_at](http://glados.ucd.ie/Coexpression/display_network.php?gene_name=55930&survival_type=dfs) | [55930](http://www.ncbi.nlm.nih.gov/gene?term=55930) | [MYO5C](http://coxpresdb.jp/data/locus/55930.shtml) | myosin VC | 0.53 | 0.85 | 5.25E-02 |
| [51158_at](http://glados.ucd.ie/Coexpression/display_network.php?gene_name=400451&survival_type=dfs) | [400451](http://www.ncbi.nlm.nih.gov/gene?term=400451) | [FAM174B](http://coxpresdb.jp/data/locus/400451.shtml) | family with sequence similarity 174, member B | 0.51 | 0.82 | 1.76E-02 |
| [221874_at](http://glados.ucd.ie/Coexpression/display_network.php?gene_name=57535&survival_type=dfs) | [57535](http://www.ncbi.nlm.nih.gov/gene?term=57535) | [KIAA1324](http://coxpresdb.jp/data/locus/57535.shtml) | KIAA1324 | 0.53 | 0.79 | 4.32E-03 |
| [212442_s_at](http://glados.ucd.ie/Coexpression/display_network.php?gene_name=253782&survival_type=dfs) | [253782](http://www.ncbi.nlm.nih.gov/gene?term=253782) | [CERS6](http://coxpresdb.jp/data/locus/253782.shtml) | ceramide synthase 6 | 0.51 | 0.94 | 4.72E-01 |
| [201596_x_at](http://glados.ucd.ie/Coexpression/display_network.php?gene_name=3875&survival_type=dfs) | [3875](http://www.ncbi.nlm.nih.gov/gene?term=3875) | [KRT18](http://coxpresdb.jp/data/locus/3875.shtml) | keratin 18 | 0.62 | 1.13 | 1.37E-01 |
| [211110_s_at](http://glados.ucd.ie/Coexpression/display_network.php?gene_name=367&survival_type=dfs) | [367](http://www.ncbi.nlm.nih.gov/gene?term=367) | [AR](http://coxpresdb.jp/data/locus/367.shtml) | androgen receptor | 0.51 | 0.86 | 7.16E-02 |
| [35148_at](http://glados.ucd.ie/Coexpression/display_network.php?gene_name=27134&survival_type=dfs) | [27134](http://www.ncbi.nlm.nih.gov/gene?term=27134) | [TJP3](http://coxpresdb.jp/data/locus/27134.shtml) | tight junction protein 3 | 0.55 | 0.93 | 4.10E-01 |
| [204067_at](http://glados.ucd.ie/Coexpression/display_network.php?gene_name=6821&survival_type=dfs) | [6821](http://www.ncbi.nlm.nih.gov/gene?term=6821) | [SUOX](http://coxpresdb.jp/data/locus/6821.shtml) | sulfite oxidase | 0.54 | 0.93 | 4.10E-01 |
| [212148_at](http://glados.ucd.ie/Coexpression/display_network.php?gene_name=5087&survival_type=dfs) | [5087](http://www.ncbi.nlm.nih.gov/gene?term=5087) | [PBX1](http://coxpresdb.jp/data/locus/5087.shtml) | pre-B-cell leukemia homeobox 1 | 0.57 | 0.97 | 6.93E-01 |
| [222212_s_at](http://glados.ucd.ie/Coexpression/display_network.php?gene_name=29956&survival_type=dfs) | [29956](http://www.ncbi.nlm.nih.gov/gene?term=29956) | [CERS2](http://coxpresdb.jp/data/locus/29956.shtml) | ceramide synthase 2 | 0.51 | 1.03 | 7.42E-01 |
| [203962_s_at](http://glados.ucd.ie/Coexpression/display_network.php?gene_name=10529&survival_type=dfs) | [10529](http://www.ncbi.nlm.nih.gov/gene?term=10529) | [NEBL](http://coxpresdb.jp/data/locus/10529.shtml) | nebulette | 0.5 | 0.94 | 4.25E-01 |
| [213412_at](http://glados.ucd.ie/Coexpression/display_network.php?gene_name=27134&survival_type=dfs) | [27134](http://www.ncbi.nlm.nih.gov/gene?term=27134) | [TJP3](http://coxpresdb.jp/data/locus/27134.shtml) | tight junction protein 3 | 0.54 | 0.99 | 8.81E-01 |
| [212151_at](http://glados.ucd.ie/Coexpression/display_network.php?gene_name=5087&survival_type=dfs) | [5087](http://www.ncbi.nlm.nih.gov/gene?term=5087) | [PBX1](http://coxpresdb.jp/data/locus/5087.shtml) | pre-B-cell leukemia homeobox 1 | 0.57 | 0.99 | 8.71E-01 |
| [210239_at](http://glados.ucd.ie/Coexpression/display_network.php?gene_name=10265&survival_type=dfs) | [10265](http://www.ncbi.nlm.nih.gov/gene?term=10265) | [IRX5](http://coxpresdb.jp/data/locus/10265.shtml) | iroquois homeobox 5 | 0.52 | 1.09 | 2.76E-01 |
| [211778_s_at](http://glados.ucd.ie/Coexpression/display_network.php?gene_name=58495&survival_type=dfs) | [58495](http://www.ncbi.nlm.nih.gov/gene?term=58495) | [OVOL2](http://coxpresdb.jp/data/locus/58495.shtml) | ovo-like 2 (Drosophila) | 0.53 | 0.91 | 2.53E-01 |
| [202525_at](http://glados.ucd.ie/Coexpression/display_network.php?gene_name=5652&survival_type=dfs) | [5652](http://www.ncbi.nlm.nih.gov/gene?term=5652) | [PRSS8](http://coxpresdb.jp/data/locus/5652.shtml) | protease, serine, 8 | 0.53 | 1.01 | 9.47E-01 |

# Supplementary Table S2. Clinicopathological parameters of breast cancer patients for TMA analysis.

# A tissue microarray (TMA) composed of duplicate cores from 144 patients with invasive breast cancer was obtained for immunohistochemical staining of JAM-A, FOXA1, HER3 and β-catenin expression. The clinicopathological parameters of the patients whose tissues were included in this TMA are shown below.

| **Parameter** | **No. Patients (n=144)** | **Total %** | **Relative %** |
| --- | --- | --- | --- |
| **Age (years)** |  |  |  |
| ≤ 50 | 20 | 13.9 |  |
| > 50 | 124 | 86.1 |  |
| Median (range) | 64.5 (34-97) |  |  |
| **Tumour Size (mm)** |  |  |  |
| ≤ 20 | 73 | 50.7 |  |
| > 20 | 71 | 49.3 |  |
| **NHG** |  |  |  |
| G1 | 22 | 15.3 |  |
| G2 | 64 | 44.4 |  |
| G3 | 58 | 40.3 |  |
| **Molecular Subtype** |  |  |  |
| Luminal A | 109 | 75.7 | 79.6 |
| Luminal B | 8 | 5.6 | 5.8 |
| HER2+ | 5 | 3.5 | 3.7 |
| Triple Negative | 15 | 10.4 | 10.9 |
| Unknown | 7 | 4.9 |  |
| **ER Status** |  |  |  |
| Negative | 19 | 13.2 |  |
| Positive | 125 | 86.8 |  |
| **PR Status** |  |  |  |
| Negative | 44 | 30.6 |  |
| Positive | 100 | 69.4 |  |
| **HER2 Status** |  |  |  |
| Negative | 130 | 90.3 | 93.5 |
| Positive | 9 | 6.3 | 6.5 |
| Unknown | 5 | 3.4 |  |

# NHG = Nottingham Histological Grade; ER = Oestrogen Receptor; PR = Progesterone Receptor

# Supplementary Table S3. Expression of JAM-A, β-catenin, FOXA1 and HER3 within the breast cancer TMA.

# A tissue microarray (TMA) composed of duplicate cores from 144 patients with invasive breast cancer was immunohistochemically stained for JAM-A, FOXA1, HER3 and β-catenin expression. Membranous expression of JAM-A in tumor cells was semi-quantitatively scored as either high (2+ and 3+) or low (0 and 1+). Similarly to JAM-A expression, β-catenin, FOXA1 and HER3 expression were stratified as high (2+ and 3+) or low (0 and 1+). β-catenin was also analysed according to its absence (score = 0) or presence in each of the cellular compartments. In all cases, cores that were unscorable due to loss of tissue or absence of tumour were classified as “unknown”.

| **Parameter** | **No. Patients (n=144)** | **Total %** | **Relative %** |
| --- | --- | --- | --- |
| **JAM-A Status** |  |  |  |
| Low | 70 | 48.6 | 55.6 |
| High | 56 | 38.9 | 44.4 |
| Unknown | 18 | 12.5 |  |
| **β-catenin Status** |  |  |  |
| Low | 40 | 27.8 | 31.0 |
| High | 89 | 61.8 | 69.0 |
| Unknown | 15 | 10.4 |  |
| **β-catenin Localization** |  |  |  |
| Membrane | 5 | 3.5 | 3.9 |
| Cytoplasm/Nucleus | 102 | 70.8 | 79.1 |
| Absent | 22 | 15.3 | 17.0 |
| Unknown | 15 | 10.4 |  |
| **FOXA1 Status** |  |  |  |
| Low | 23 | 16.0 | 18.3 |
| High | 103 | 71.5 | 81.7 |
| Unknown | 18 | 12.5 |  |
| **HER3 Status** |  |  |  |
| Low | 73 | 50.7 | 59.8 |
| High | 49 | 34.0 | 40.2 |
| Unknown | 22 | 15.3 |  |

# Supplementary Table S4. Association of JAM-A expression with FOXA1 and HER3 expression with TMA features.

# We examined the relationship between high versus low expression of JAM-A and that of FOXA1 and HER3. Curiously, high JAM-A expression was not significantly associated with high FOXA1 or HER3 expression; a fact that likely reflects the limited size and molecular subtype distribution of this TMA.

| **Variable** | **N** | **High JAM-A** | **%** | **Low JAM-A** | **%** | **p-value** |
| --- | --- | --- | --- | --- | --- | --- |
| **FOXA1 Expression** | 124 |  |  |  |  |  |
| Low |  | 18 | 32.1 | 5 | 7.4 | <0.001* |
| High |  | 38 | 67.9 | 63 | 92.6 |  |
| **HER3 Expression** | 122 |  |  |  |  |  |
| Low |  | 38 | 67.9 | 35 | 53.0 | 0.138 |
| High |  | 18 | 32.1 | 31 | 47.0 |  |

# * p<0.05 by two-sided Fisher’s Exact Test or Chi Square Test.
